# Selection of DNA Aptamers for Subcellular Localization of RBSDV P10 Protein in the Midgut of Small Brown Planthoppers by Emulsion PCR-Based SELEX

**DOI:** 10.3390/v12111239

**Published:** 2020-10-30

**Authors:** Haoqiu Liu, Yijun Zhou, Qiufang Xu, Sek-Man Wong

**Affiliations:** 1 Department of Biological Sciences, National University of Singapore, Singapore 117543, Singapore; liuhaoqiu@u.nus.edu; 2 National University of Singapore (Suzhou) Research Institute, Suzhou 215123, China; 3 Key Laboratory of Food Quality and Safety of Jiangsu Province, State Key Laboratory Breeding Base, Institute of Plant Protection, Jiangsu Academy of Agricultural Sciences, Nanjing 210014, China; yjzhou@jaas.ac.cn; 4 Temasek Life Sciences Laboratory, 1 Research Link, Singapore 117604, Singapore

**Keywords:** aptamers, rice black-streaked dwarf virus, RBSDV, SELEX, emulsion PCR

## Abstract

Rice black-streaked dwarf virus (RBSDV), classified under the *Reoviridae, Fijivirus* genus, caused an epidemic in the eastern provinces of China and other East Asian countries and resulted in severe yield loss in rice and wheat production. RBSDV is transmitted by the small brown planthopper (SBPH, *Laodelphax striatellus* Fallén) in a persistent manner. In order to provide a stable and cost-effective detection probe, in this study we selected three DNA aptamers (R3, R5 and R11) by an optimized, standardized and time saving emulsion PCR-based SELEX, for the detection of RBSDV outer-shell P10 protein for in situ localization studies in the midgut of SBPH. The specificity of these three DNA aptamers was tested through detection of the P10 protein using an enzyme-linked oligonucleotide assay (ELONA) and aptamer-based dot-blot ELISA. All three DNA aptamers can be used to detect RBSDV P10 protein by immunofluorescent labeling in the midgut of RBSDV-infected SBPH. These data show that the selected aptamers can be used for the detection of RBSDV P10 protein in vitro and in vivo. This is the first report of aptamers being selected for detection of a rice virus capsid protein.

## 1. Introduction

Rice black-streaked dwarf virus (RBSDV) belongs to the *Reoviridae* Family, *Spinareovirinae*, *Fijivirus* genus [1,2], causing rice black-streaked dwarf disease and maize rough dwarf disease [3,4]. RBSDV is transmitted by the small brown plant hopper (SBPH, *Laodelphax striatellus* Fallén) in a persistent propagative manner and infects many plant species in Gramineae including rice, maize, wheat and barley [1,2]. Typical symptoms of infected rice plants include stunting, darkening of leaf color, and tumors on leaves and stems. The infected rice plants fail to produce heads when infection is established at the seedling stage. When infection occurs at the tillering stage, the panicles are usually closed, causing severe loss of crop production [5,6]. In 2013, a field survey confirmed that 333 out of 915 plants from five provinces surveyed in China were infected with RBSDV [7].

RBSDV is a double-layered icosahedral virus (75–80 nm in diameter) comprised of 10 dsRNA segments (S1–S10) and 6 structural proteins which are P1, P2, P3, P4, P8 and P10 in its viral particle [8]. RBSDV P10 protein is the major outer capsid protein [9]. It is used as an indicator of RBSDV infection in disease diagnosis [7,10]. The current techniques for RBSDV are based on detection of the gene sequences or viral proteins. For detection of RBSDV, several methods have been established, such as reverse transcription polymerase chain reaction (RT-PCR) [8], Northern blotting [8,11,12], quantitative real time polymerase chain reaction (qRT-PCR) [13], RT-loop-mediated isothermal DNA amplification (RT-LAMP) [14], single small brown plant hopper RT-PCR [15] and reverse transcription-recombinase polymerase amplification (RT-RPA) [16]. As for detection of RBSDV in situ by immunofluorescent labeling, antibodies against RBSDV P7-1 [17], P9-1 [17] and P10 [18] have been adopted for localization of RBSDV in the SBPH midgut. However, high cost culture reagents and equipment are required for these methods. Therefore, we aimed to select a few DNA-based probes as alternatives for detection of RBSDV P10 in its transmission vector SBPH for a subcellular localization study of virus–vector interactions.

**Figure 1:**
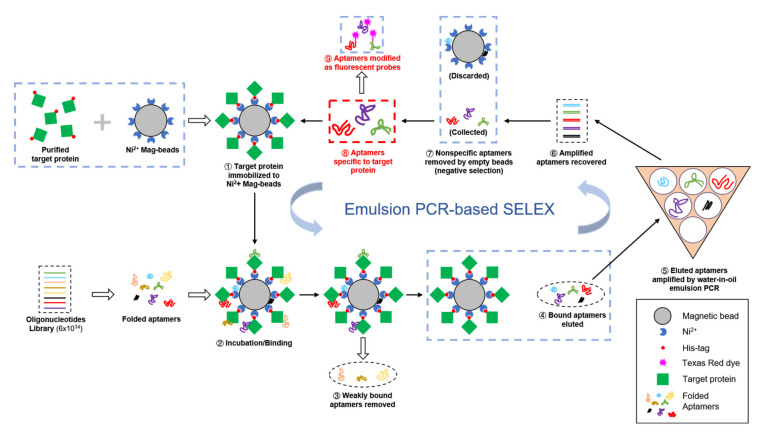
Selection of aptamers against target viral protein by Systematic Evolution of Ligands by Exponential Enrichment (SELEX) with emulsion PCR.

Aptamers are single-strand DNA or RNA with a length of 56–120 nt comprised of a variable region of 20–80 nt flanked by fixed primer sequences. They form secondary structures such as hairpin loops, internal loops, bulge loops, multibranch loops or pseudoknots. Aptamers can bind to a great variety of targets including metal ions, small molecules, proteins, lipids or cells. Single-strand nucleotide aptamers are thermally stable and can be easily modified and applied to ELISA assays, immunofluorescent labeling, aptamer-pulldown assays, flow cytometry and drug delivery. The small molecular size of the aptamer enhances its accessibility to target molecules, which enables it to function as a probe more effectively [19]. As depicted in Figure 1, to select a specific and sensitive aptamer for the target, the vast diversity of random sequences in the variable region of the aptamers will be affinity-based selected through a process called Systematic Evolution of Ligands by Exponential enrichment (SELEX) [20,21]. During each round of the affinity selection, unbound ssDNA aptamers are washed away and the ssDNA aptamers bound to immobilized target molecules are collected and amplified in PCR to serve as the DNA library for the next round of selection. Finally, ssDNA aptamers with a high affinity to target molecules are enriched after 10 rounds of SELEX and the sequence of candidate DNA aptamers is determined.

The screening of a ssDNA/RNA aptamer library by SELEX in each round involves the PCR amplification of the eluted aptamers for the next round of selection. In order to maintain the relative abundance of each eluted aptamer in the library before and after PCR, despite the high complexity of the template mixture, a uniform amplification is necessary. PCR techniques such as BEAMing (beads, emulsion, amplification, and magnetics) [22] and emulsion PCR [23] provided valuable approaches to meet the requirements of library amplification in SELEX. The key to uniform amplification is the physical partitioning of each template into individual micelles prior to PCR amplification, thus avoiding PCR bias [24] and hybridized by-product formation [25,26] which have been observed when a high complexity template mixture is amplified in conventional PCR. In this study, the concept of emulsion PCR combined with the formula of the organic phase used in BEAMing for emulsification was adopted and improved to amplify enriched aptamers in SELEX.

Aptamers have already been developed to detect animal and human viruses. DNA aptamers selected against Influenza A virus subtype H1N1 virus particles have been successfully applied in virus diagnosis [27]. Similar cases for other animal viruses such as Avian influenza virus H5N1 [28], Muscovy duck parvovirus [29], Respiratory syncytial virus [30], Human noroviruses [31,32,33], Hepatitis B virus [34], Hepatitis C virus [35] and Bovine viral diarrhea virus [36] have been reported. However, there is only one example of plant virus detected by aptamers, the detection of Apple stem pitting virus [37]. In this study, we aimed to select DNA aptamers to detect the plant virus RBSDV P10 in the insect vector SBPH using an improved emulsion PCR-based SELEX method. We also exploited the advantage of the small molecular size of aptamers which brings a greater accessibility to target molecules when entering the subcellular compartments in immunofluorescent labelling for studies of virus–vector interactions.

## 2. Materials and Methods

### 2.1. Expression and Purification of RBSDV His-P10 Protein

The ORF of RBSDV S10 gene was inserted into the pET-15b vector which contained a 6×His-tag at the N terminus of the P10 protein. The recombinant vector pET-15b-His-P10 was transformed into *E. coli* BL21-CodonPlus (DE3)-RIPL cells for expression at 16 °C with 0.6 mM IPTG and shaken at 120 rpm for 16 h. Cells were pelleted by centrifugation at 5000× *g* for 20 min and sonicated in the lysis buffer (50 mM Tris-HCl, 0.2 M NaCl, 5% glycerol, 1% Triton X-100) and centrifuged at 12,000× *g* for 20 min at 4 °C. The supernatant was collected and the RBSDV His-P10 protein was recovered from the lysate by Immobilized Metal Affinity Chromatography (IMAC) with the Ni-NTA agarose (QIAGEN Cat No./ID: 30210, Hilden, NRW, Germany), following the manufacturer’s protocol. The eluted protein was concentrated by a 30-kDa concentrator (Amicon Ultra-15 30K devices, Merk Millipore, Burlington, MA, USA). The purity of concentrated proteins was verified by 12.5% SDS-PAGE, followed by Coomassie Brilliant Blue R250 staining and the identity of the purified protein was confirmed by the RBSDV P10 mAB [7] (generously donated by Prof. Jianxiang Wu from Zhejiang University, Hangzhou, Zhejiang, China) in Western blot. The RBSDV His-P10 protein was vacuum dried after dialysis to remove NaCl and dissolved in the 1× SELEX buffer (140 mM NaCl, 2 mM KCl, 5 mM MgCl_2_, 2 mM CaCl_2_, 20 mM Tris-HCl, pH 7.4, 0.05% Tween 20) for short-term storage at 4 °C.

### 2.2. Selection of Putative Aptamers against RBSDV His-P10 Protein by Emulsion PCR-Based SELEX

#### 2.2.1. Construction of the N80 ssDNA Library for Putative Aptamers Selection

The N80 ssDNA library was synthesized as a sequence of 80 nt continuous random ssDNA (N80) flanked by a pair of fixed primer sequences as 5′-TGACACCGTACCTGCTC-N×80-AAGCACGCCAGGGACTAT-3′. The N80 ssDNA library and primers (For 5′ end primer: 5′-TGACACCGTACCTGCTCT-3′, and for 3′ end primer: 5′-ATAGTCCCTGGCGTGCTT-3′) were diluted to 100 mM as stock solutions. The primer set, devoid of hairpin, dimer and heterodimer-forming sequences, was developed earlier [38] and subsequently widely adopted for SELEX selections.

#### 2.2.2. Immobilization of RBSDV His-P10 Protein to Ni-NTA Magnetic Resin

The RBSDV His-P10 protein was diluted to 1 μg/μL in the 1× SELEX buffer. The RBSDV His-P10 protein (30 μL) was mixed with 30 μL (1:1 *v*/*v* suspension) of pre-equilibrated Ni-NTA magnetic beads (Thermofisher HisPur™ Ni-NTA Magnetic Beads, Cat: 88831, Waltham, MA, USA). The binding was conducted at 25 °C for 1 h with inversion of the mixture in a 1.5-mL centrifuge tube every 5 min to prevent aggregation of beads. The RBSDV His-P10-beads complex was washed three times with 500 μL of the 1× SELEX buffer before the aptamer selection.

#### 2.2.3. Emulsion PCR (ePCR)

For the ssDNA library, 50 μL of the ssDNA eluted from each selection round was prepared as DNA templates in 200 μL of the aqueous PCR phase containing 68 μL H_2_O, 20 μL 10× PCR buffer (100 mM Tris-HCl (pH 8.8 at 25 °C), 500 mM KCl), 32 μL 25 mM MgCl_2_, 8 μL 100 mM 5′ primer, 8 μL 100 mM 3′ primer, 8 μL 10 mM dNTP, 2 μL BSA (1 mg/mL), 50 μL eluted ssDNA and 4 μL Taq DNA polymerase (5 u/μL).

For the preparation of 1200 μL of the organic phase [22], 880 μL Tegosoft DEC (Evonik Degussa, Essen, Germany), 80 μL mineral oil (Sangon Biotech, Shanghai, China) and 240 μL ABIL WE 09 (Evonik Degussa) were added into a 1.5-mL centrifuge tube, followed by vortexing it thoroughly.

For the ePCR, 50 μL of the aqueous phase of the PCR mixture was added to 300 μL of the organic phase in a 2-mL centrifuge tube, followed by emulsification with vortexing for 5 min at 4 °C to form droplets with an average diameter of 10 μm. Before PCR, 50 μL of emulsified PCR mixture was aliquoted into each 0.2 mL PCR tubes. The ePCR program was set up as (95 °C, 2 min; (95 °C 30 s, 55 °C 60 s, 72 °C 3 min) for 20, 15 or 10 cycles (please refer to Table 1); 72 °C 5 min).

For the DNA recovery, all emulsions were pooled into a 1.5-mL centrifuge tube and vortexed with 1 mL water-saturated isobutanol for 1 min to break the emulsion, followed by 1 min of centrifugation at 12,000×* g* at 25 °C. The organic phase was then discarded and 0.5 mL hydroxybenzene-chloroform-Isoamyl alcohol (25:24:1, pH 7.8) was added to the aqueous phase, followed by vortexing for 10 s. The mixture was centrifuged at 12,000× *g* for 1 min and the aqueous phase was collected. The dsDNA in the aqueous phase was recovered by QIAquick PCR Purification Kit (Qiagen 28104, Germany), following the manufacturer’s protocol.

#### 2.2.4. Optimization of Emulsification, Concentrations of MgCl_2_ and BSA of ePCR

To compare the methods reported for the emulsification [22,39], the uniformity of aqueous droplets in emulsions created by the tissue lyser (30 Hz for 20 s, 30 Hz for 20 s, 60 Hz for 20 s and 60 Hz for 40 s, respectively) and vortexing (at 4 °C or 25 °C) was compared using a light microscope.

For the optimization of MgCl_2_ and BSA concentrations in ePCR, 50 μL aqueous phase of the ePCR mix containing 5 μL of 10× PCR buffer, 2 μL 100 μM 5′ primer, 2 μL 100 μM 3′ primer, 2 μL 10 mM dNTP and Taq (5 u/μL), 10 μL N80 ssDNA library (250 pM) with gradient MgCl_2_ (final concentration from 0.5 to 5 mM) or BSA (final concentration from 0 to 1 mg/mL and 10 uL 25 mM MgCl_2_) were emulsified with 300 μL of the mixed organic phase and amplified for 20 cycles.

#### 2.2.5. Selection of Putative Aptamers against RBSDV His-P10 Protein by Emulsion PCR-Based SELEX

The selection of DNA aptamers for the RBSDV His-P10 protein was conducted by the emulsion PCR-based-SELEX with detailed parameters recorded in Table 1. In order to allow adequate ssDNA for the SELEX screening, 1 nmol of N80 ssDNA library (35.7 μg, 6.02 × 10^14^) was used in the first round of SELEX. Briefly, the DNA library was denatured in 500 μL of the 1× SELEX buffer at 95 °C for 10 min, vortexed for 2 s and placed in an ice-water bath immediately for 10 min to allow ssDNA folding. The folded ssDNA was then equilibrated at 25 °C for 10 min and incubated with the immobilized His-P10-Ni-NTA-beads at 25 °C (for incubation time please refer to Table 1) with intermittent tube inversions. After the unbound ssDNA was washed away by the 1× SELEX buffer, the ssDNA bound to the His-P10-Ni-NTA-beads was eluted by incubation with 100 μL H_2_O at 95 °C for 5 min, followed by separation on a magnetic stand. The eluted ssDNA was used as the templates in ePCR for uniform amplification. The DNA output yield from the ePCR of each round was used as an index to determine the stringency of the next round. Briefly, the parameters of stringency (input DNA, amount of protein, binding time, wash frequency and time, and ePCR cycles, Table 1) of the next round were adjusted to increase when the DNA output yield from the ePCR exceeded 3 μg. When it was lower than 3 μg, the stringency for the next round would be maintained to expand the DNA library. In case it was lower than 1 μg, a scale-up ePCR would be conducted to amplify directly from the recovered ePCR product. In the final round, the most stringent parameters were achieved.

To prevent any enrichment of non-specific binders, the folded DNA library of aptamers for the RBSDV His-P10 protein was negatively selected against 20 μL clean equilibrated Ni-NTA magnetic beads before SELEX round 7 and round 9. The supernatant after the magnetic beads separation was collected as the input DNA for round 7 and 9, respectively.

For the analysis of the quality of the DNA output after the ePCR, 0.1 μg of the output DNA from each round of SELEX was separated using 2% agarose gel electrophoresis and stained by ethidium bromide (0.5 µg/mL). For confirmation of the sequences of enriched aptamers, 0.3 pmol ePCR product from round 10 was ligated with 0.01 pmol pMD18T vector (TAKARA BIO INC., Japan) and 50 random clones were sequenced. The constructs harboring primer-polymers or repeated sequences were removed and the non-redundant sequences were selected (Table 2). The full sequences of 23 F-aptamers (from forward strands) and 23 R-aptamers (from reverse strands) were aligned by ClustalW in MEGA5 software for the generation of Neighbor-joining trees.

### 2.3. Verification of Putative Aptamers by ELONA, Aptamer-Based Dot-Blot ELISA, and Immunofluorescent Localization of RBSDV P10 Protein

#### 2.3.1. Selection of Putative Aptamers against RBSDV His-P10 Protein Measured by Enzyme-Linked Oligonucleotide Assay (ELONA)

The biotinylated or Texas-Red-tagged 5′ primer or 3′ primer was used to synthesize the putative aptamer probes from the sequenced recombinant pMD18T-aptamer constructs using conventional PCR with the following components: 1 ng vector template, 1× PCR buffer, 1.5 mM MgCl_2_, 100 μM dNTP, 1 u Taq polymerase, 0.4 μM biotinylated or Texas-Red-tagged 5′ primer and 0.4 μM conventional 3′ primer for F-aptamer synthesis; or 0.4 μM conventional 5′ primer and 0.4 μM biotinylated or Texas-Red-tagged 3′ primer for R-aptamer synthesis. The program was set (95 °C, 2 min; (95 °C 30 s, 55 °C 60 s, 72 °C 1 min) for 20 cycles; 72 °C 5 min). PCR products were designated as F-aptamers (modified on forward strand) or R-aptamers (modified on reverse strand). The dsDNA purified by ethanol precipitation were diluted to 1 μg/mL by the 1× SELEX buffer and denatured to single strands as described for folding into aptamers.

The enzyme-linked oligonucleotide assay (ELONA) was conducted following methods previously described [40]. Briefly, 200 ng of the RBSDV His-P10 protein in the coating buffer (44 mM sodium bicarbonate, 6 mM sodium carbonate, pH 9.6) were coated onto an ELISA plate overnight at 4 °C, followed by washing three times with 200 μL washing buffer (50 mM Tris, 0.14 M NaCl, 0.05% Tween 20, pH 8.0). For blocking the wells, 100 μL 3% (*w*/*v*) BSA (50 mM Tris, 0.14 M NaCl, 3% BSA, pH 8.0) were added to each well at 25 °C for 1 h, followed by washing three times with 200 μL washing buffer. Then 100 μL (1 μg/mL) of the biotinylated aptamer were added to corresponding wells and incubated at 25 °C for 1 h with gentle shaking at 70 rpm. Wells coated by BSA alone were used as a negative control. After washing three times with 200 μL of the 1× SELEX buffer, 100 μL 0.2 μg/mL HRP-streptavidin dissolved in the 1× SELEX buffer were added to each well and incubated at 25 °C for 1 h with gentle shaking at 70 rpm. After washing five times with the 1× SELEX buffer, 100 μL TMB (EL-TMB Chromogenic Reagent kit, Sangon Biotech, Shanghai, China) were added into each well for reaction at 25 °C for 5 min in the dark. To stop the reaction, 50 μL 0.5 M H_2_SO_4_ was added into each well. The absorbance at 450 nm was measured by an ELISA microplate reader (MULTISKAN MK3, Thermo Scientific, Waltham, MA, USA).

#### 2.3.2. Aptamer-Based Dot-Blot ELISA for Verification of Aptamer Specificity

For the sample preparation, cytosolic proteins from 100 mg of RBSDV-infected or RBSDV-free SBPH were extracted (Membrane and Cytosol Protein Extraction Kit P0033, Beyotime Biotechnology, Shanghai, China) following manufacturer’s instructions. For the verification of the detection limit of aptamers, 1 μL out of 1 mL extracted cytosolic protein fractions was diluted into 10^2^×, 10^3^×, 10^4^×, 10^5^× and 10^6^×, and 1 μL each of the diluted proteins was dot-blotted onto the nitrocellulose (NC) membrane (0.45 μm) and blocked by 3% BSA in the 1× SELEX buffer for 1 h at 25 °C with 70 rpm gentle shaking. Three milliliters of folded biotinylated ssDNA aptamer (1 μg/mL, as described in Section 2.3.1) in the 1× SELEX buffer were incubated with the NC membrane overnight at 25 °C with 70-rpm gentle shaking. The NC membranes were then washed 3 times with the 1× SELEX buffer for 5 min each before incubating with 0.2 μg/mL HRP-streptavidin in the 1× SELEX buffer at 25 °C for 1 h with 70 rpm gentle shaking, followed by 3 times washing with the 1× SELEX buffer for 5 min each. The signals on membranes were developed using the Clarity Western ECL Substrate Kit (Bio-Rad, 1705060, Hercules, CA, USA), following the manufacturer’s instructions. The RBSDV P10 protein mAB (1 μg/mL) [7] was used as the positive control probe.

#### 2.3.3. Targeting RBSDV P10 Protein by Fluorescent Aptamers in the Midgut of RBSDV-Infected *L. striatellus*

The Texas-Red-tagged aptamers R3, R5 and R11 were tested for their binding to the RBSDV P10 protein in the RBSDV-infected SBPH midgut. The Texas-Red-tagged aptamer R1 was used as a negative control. Similarly, the anti-RBSDV P10 protein mAB, together with the FITC-conjugated rabbit anti-mouse IgG were used as a positive control. The midgut of virus-free SBPHs was used as a negative control. Briefly, the midguts of SBPH were dissected in the phosphate saline buffer (12 mM phosphate buffer, 137 mM NaCl, pH 7.4) and fixed in 4% polyformaldehyde for 5 min, followed by washing three times in the 1× SELEX buffer, permeabilized by 2% Triton X-100 in the 1× SELEX buffer for 30 min and blocked by 3% BSA in the 1× SELEX buffer for 1 h at 25 °C. The dissected midgut was incubated with 2 μg/mL mAB against RBSDV P10 protein with 1 μg/mL Texas-Red-tagged aptamer ssDNA (as described in Section 2.3.1) in the 1× SELEX buffer at 4 °C overnight. After washing three times, 1:5000 diluted FITC-labeled rabbit anti-mouse IgG were added and incubated for 1 h at 25 °C, followed by washing three times with the 1× SELEX buffer. The localized fluorescence of FITC and Texas-Red were excited at 488 nm and 594 nm under a confocal microscope (Zeiss LSM 710, Oberkochen, BW, Germany), respectively.

#### 2.3.4. Preparation of RBSDV-Free and RBSDV-Infected SBPH

The SBPH used in this study was reared inside the incubation chamber in the Institute of Plant Protection, Jiangsu Academy of Agricultural Sciences (JAAS, Nanjing, China). The nymphs of SBPH at the second stage fed on either RBSDV-free or RBSDV-infected rice plants (*Oryza sativa* subsp. *Japonica* variety “Huaidao 5”) for 3 d and then transferred to RBSDV-free rice seedlings (*O. sativa* subsp. *Japonica* variety “Wuyujing 3”) for an additional 7 d with a photoperiod of 16:8 h (light:dark) at 25 ± 3 °C and 55 ± 5% relative humidity as described previously [41].

## 3. Results

### 3.1. Expression and Purification of RBSDV His-P10 Protein

**Figure 2:**
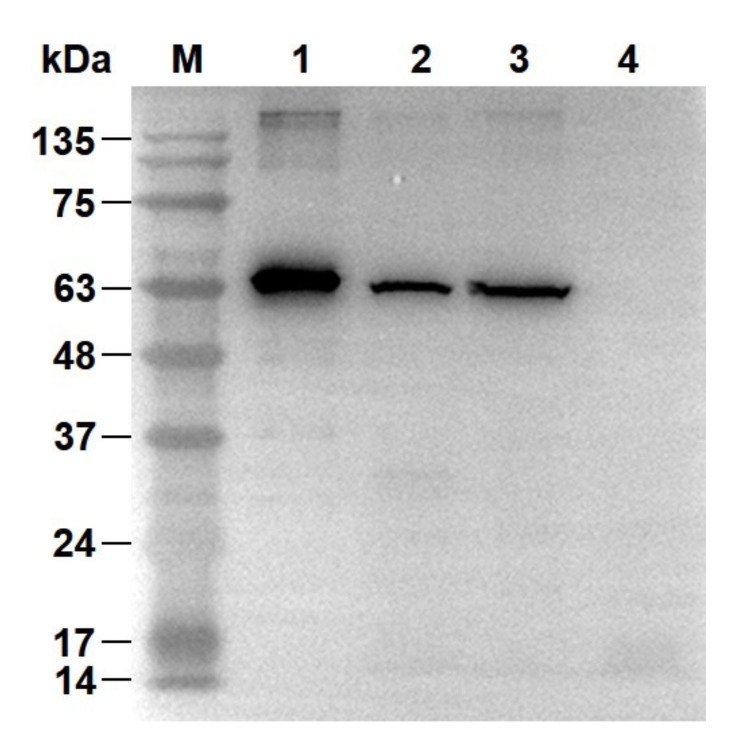
Confirmation of the purified rice black-streaked dwarf virus (RBSDV) His-P10 protein by the anti-P10 protein mAB. Purified RBSDV His-P10 protein (as positive control, Lane 1); membrane (Lane 2) and cytosolic (Lane 3) protein fractions of RBSDV-infected small brown planthopper (SBPH); cytosolic protein fraction of RBSDV-free SBPH (as negative control, Lane 4).

A variety of fusion tags to the RBSDV P10 protein were screened for expression (Figure S1A,B). A plasmid construct of the RBSDV His-P10 protein expressed in *E. coli* BL21-CodonPlus (DE3)-RIPL cells was selected. A band about 65 kDa was visible after induction with IPTG. The expressed protein size was consistent with the calculated molecular weight of 65.3 kDa (Figure S2A). The RBSDV His-P10 protein purified by IMAC was analyzed by SDS-PAGE and CBB-R250 staining. The results showed that there were no other visible protein bands (Figure S2B). The Western blot was also carried out to confirm the identity of the purified His-P10 protein using the anti-RBSDV P10 mAB. The purified expressed RBSDV P10 protein was used as a positive control. The membrane and cytosolic protein fractions of the RBSDV-infected SBPH were successfully detected. No band was detected in the negative control (Figure 2). These results confirmed the identity of the purified RBSDV His-P10 protein.

### 3.2. Enrichment of Putative Aptamers by Emulsion PCR-Based SELEX against RBSDV His-P10 Protein

#### 3.2.1. Optimization of ePCR

According to the comparison of uniformity of droplets under the light microscope, the emulsion created by vortexing 50 μL aqueous phase with 300 μL organic phase at 4 °C for 5 min was determined to be the best method for emulsification (Figure S3A). Droplets with a diameter of 10 μm were evenly formed as a suspension in the organic phase (Figure S3A panel E) to separate ssDNA templates into independent aqueous droplets. There were estimated to be 0.63 fmol (3.8 × 10^8^) droplets/200 μL PCR aqueous phase. Therefore, it is deduced that 22.7 pg eluted ssDNA aptamers in 200 μL aqueous phase could be amplified in the isolated droplets after the ePCR.

**Figure 3:**
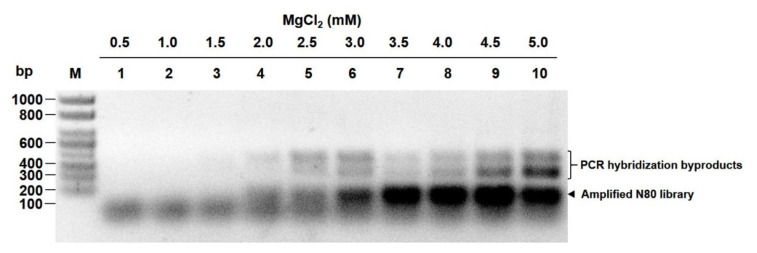
Optimization of MgCl_2_ concentration for efficient emulsification PCR (ePCR). Purified DNA from ePCR with gradient MgCl_2_ concentrations in lanes 1–10: 0.5, 1, 1.5, 2, 2.5, 3, 3.5, 4, 4.5 and 5 mM, respectively.

After the optimization of ePCR parameters, the N80 ssDNA library was successfully amplified by ePCR with 3.5 to 4 mM MgCl_2_ (Figure 3) with the least hybridization byproducts formed and the optimal concentration of BSA in ePCR was confirmed to be in the range of 0.01 to 0.1 mg/mL (Figure S3B).

#### 3.2.2. Selection of Putative Aptamers by Emulsion PCR-Based SELEX

**Figure 4:**
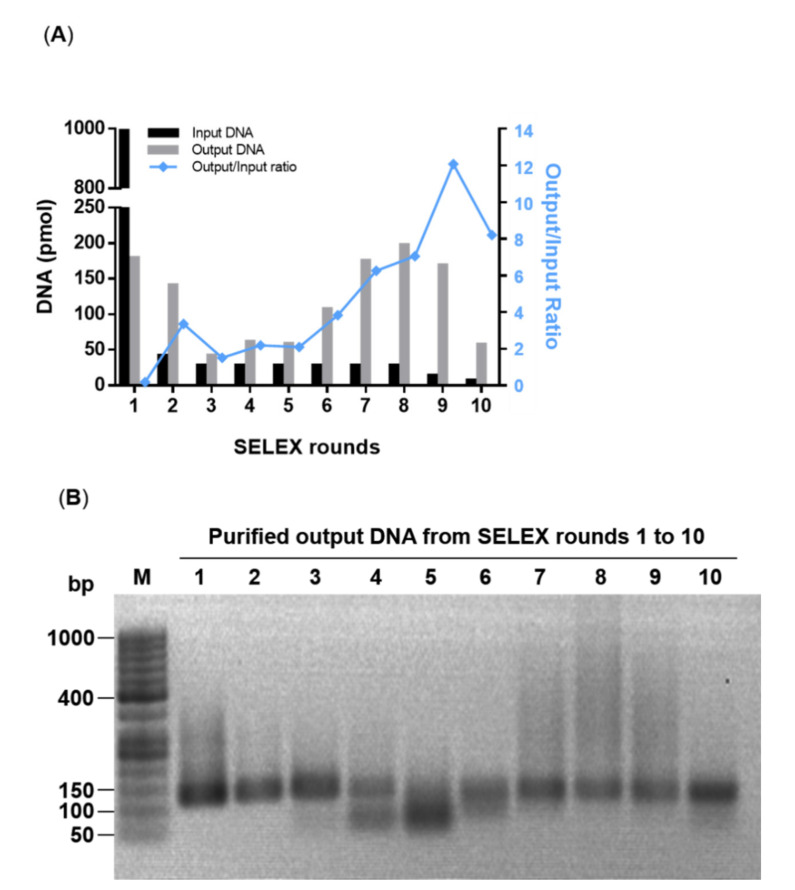
Enrichment of putative aptamers against the RBSDV His-P10 protein in SELEX. (**A**) The amount (pmol) of input DNA and output DNA in SELEX rounds 1 to 10 were plotted on the left *Y*-axis (as a bar graph); the DNA output/input ratio was plotted on the right *Y*-axis (as a line graph in blue color). (**B**) Purified output DNA from SELEX rounds 1 to 10 were separated by 2% agarose gel electrophoresis and stained by ethidium bromide (0.5 µg/mL).

The parameters of stringency in the selection of putative aptamers against the RBSDV His-P10 protein by the emulsion PCR-based-SELEX are listed in Table 1. The stringency was gradually increased from rounds 1 to 10 and the most stringent condition was achieved in round 10, which was with 7 pmol input DNA, 5 μg RBSDV His-P10 protein, 15 min of binding at 25 °C, washing three times with 1 mL 1× SELEX buffer and 10 cycles of ePCR. By this gradual increase in stringency, the output DNA amount never dropped below the minimum threshold of 3 μg, below which a repeat or scale-up round would be required. The enrichment of putative aptamers during the selection was observed at round 6 as the curve of output/input DNA amount started to increase (Figure 4A). Despite the continuous decrease in the input DNA amount in each round, the output/input DNA curve started to increase from rounds 6 to 9, up to 12.08 fold, indicating the putative aptamers possessing affinity to the RBSDV His-P10 protein were enriched.

A decrease in the amount of the recovered output dsDNA was observed from rounds 1 to 5 and an increase in primer dimers from ePCR was observed in rounds 4 and 5 (Figure 4B). However, as the putative aptamers were gradually enriched with an increasing stringency from rounds 6 to 10, the primer dimers were removed from the N80 DNA library. Although the output/input ratio of the round 10 decreased compared to round 9, the selection stringency in round 10 including input DNA, P10-beads complex, binding time and ePCR cycles were all strengthened. Therefore, ePCR products from round 10 were T-A cloned into the linearized pMD18T vector, and 50 clones were selected, of which 23 non-redundant aptamer templates with a size range of 91 to 127 bp were obtained (Table 2).

**Figure 5:**
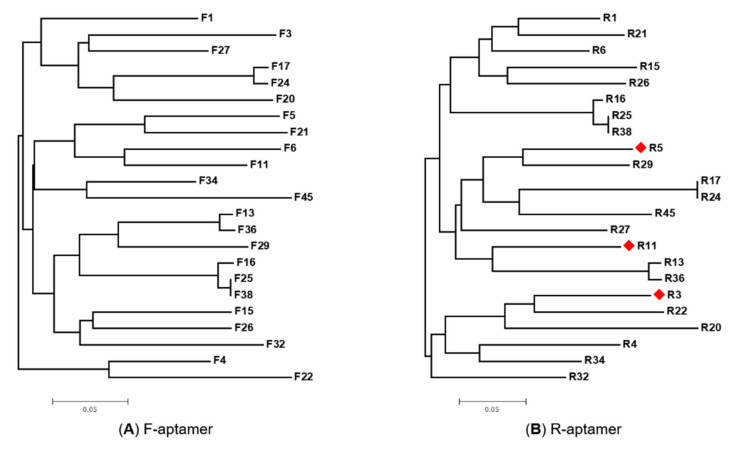
Phylogenetic analyses of 23 selected forward strand aptamers (F-aptamer) and 23 selected reverse strand aptamers (R-aptamer) against the RBSDV P10 protein. Neighbor-joining phylogenic trees of the similarity among 23 F-aptamers (**A**), and 23 R-aptamers (**B**), respectively.

Phylogenetic analyses of F-aptamers and the R-aptamers were made by ClustalW in MEGA5 software and showed that putative aptamers enriched from the N80 ssDNA library were very diverse (Figure 5). It indicated that the ssDNA library used to generate aptamers for selection is sufficiently complex. A library of 1 nmol (6.02 × 10^14^) random ssDNA aptamers was large enough to generate sufficient putative aptamers against the RBSDV His-P10 protein from the emulsion PCR-based SELEX screening.

### 3.3. Detection of RBSDV His-P10 Protein Using the Selected Putative Aptamers

**Figure 6:**
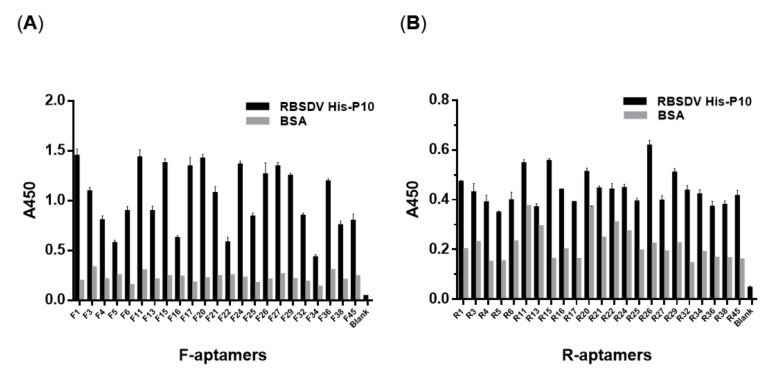
Detection of the RBSDV His-P10 protein using 23 selected F-aptamers and 23 selected R-aptamers by enzyme-linked oligonucleotide assay (ELONA). Each F-aptamer (**A**) or R-aptamer (**B**) was used at 100 μL (1 μg/mL) to detect 200 ng RBSDV His-P10 protein-coated wells using Costa EIA plates.

A total of 46 biotinylated aptamers, containing 23 F-aptamers and 23 R-aptamers, were synthesized based on the 23 TA-cloned constructs from round 10. The biotinylated aptamers were used to detect the RBSDV His-P10 protein coated in bottom wells of the ELISA plate by the enzyme-linked oligonucleotide assay (ELONA). The wells coated by BSA were used as the negative controls. The detection results were measured by reading the absorbance at 450 nm filter after the TMB-HRP reaction. The sensitivity of aptamer binding to the RBSDV His-P10 protein was positively correlated with the signal intensity (black bars) which represented the relative amount of aptamers bound to the His-P10 coated wells (Figure 6A,B). The affinity of selected aptamers varied and the affinity of F-aptamers was higher than that of R-aptamers. The A450 nm of P10/BSA ratio varied between 1.26 (aptamer R13) and 7.55 (aptamer F17), suggesting that the specificities of these 46 putative aptamers were different. The comparison between F-aptamers to the RBSDV His-P10 protein and R-aptamers to the RBSDV His-P10 protein suggested that aptamers synthesized based on the forward strands gained more affinity to the RBSDV His-P10 protein than aptamers generated from the reverse strands.

**Figure 7:**
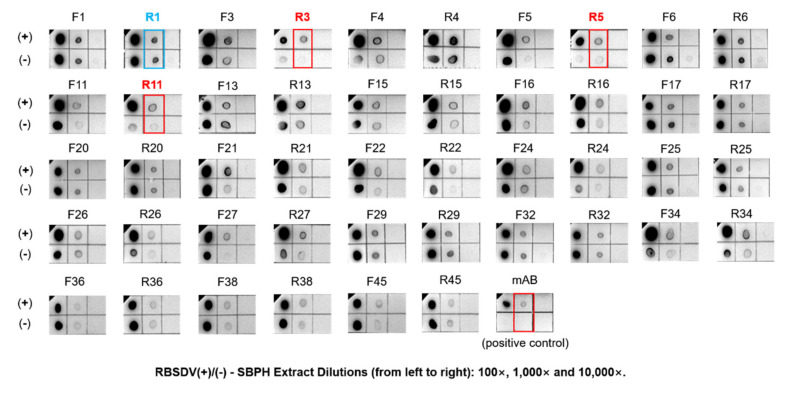
Detection of the RBSDV P10 protein with 46 biotinylated putative aptamers by Dot-ELISA. Total proteins (1 mL) extracted from either 100 mg RBSDV-infected small brown planthoppers, as SBPH (+) or RBSDV-free SBPH (−), were serially diluted, from left to right panels in each block, 100, 1000 and 10,000 times, respectively. An amount of 1 μL each of the 3 dilutions from RBSDV-infected SBPH (+) proteins (upper 3 blocks) and RBSDV-free SBPH (−) proteins (lower 3 blocks) were dot-blotted and detected by 1 μg/mL each of putative aptamers. The anti-RBSDV P10 protein monoclonal antibody (mAB, 1 μg/mL) was used as a positive control probe to detect the RBSDV P10 protein. The R1 aptamer was selected as a negative probe and aptamers R3, R5 and R11 were selected as positive probes to detect the RBSDV P10 protein in RBSDV(+)-SBPH midgut subcellular localization experiments.

The aptamer-based dot-blot ELISA assay showed that R3, R5 and R11, out of the 46 aptamer candidates, were able to detect the RBSDV P10 protein in RBSDV infected SBPH (Figure 7, each panel was cropped and grouped to allow a more convenient comparison. For uncropped figures, please refer to the Supplementary Figure S4). The other aptamers exhibited non-specific bindings to SBPH proteins. The binding assays using aptamers R3, R5 and R11 were successful even when protein samples were diluted to 1000 times (Figure 7). The detection sensitivity of R3, R5 or R11 aptamers (each at 1 μg/mL) was higher than that of anti-RBSDV P10 mAB (1 μg/mL), as the dot intensity of all three aptamers was significantly stronger than that of the positive control samples probed by anti-RBSDV P10 mAB.

### 3.4. Detection of RBSDV P10 Protein In Vivo Using the Selected Aptamers by Immunofluorescence

**Figure 8:**
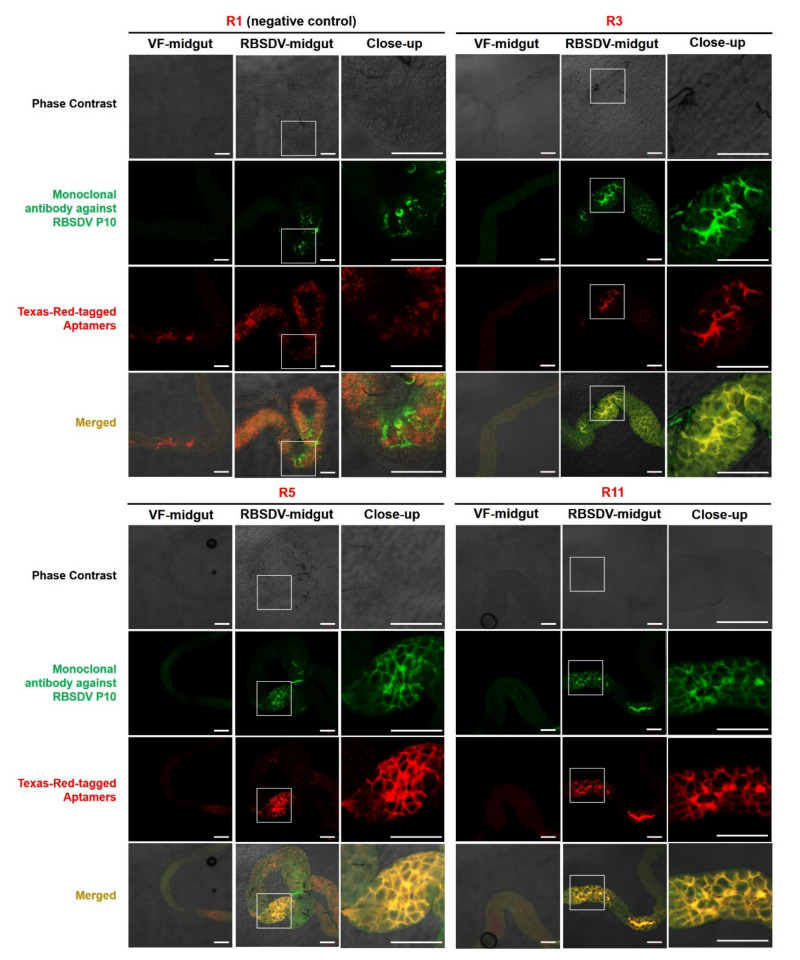
Detection of the RBSDV P10 in the midgut of RBSDV-infected small brown planthoppers (SBPH) using the RBSDV P10 protein monoclonal antibody and Texas-Red-tagged aptamers R1 (negative control), R3, R5 and R11. The localization of RBSDV P10 by the anti-P10 monoclonal antibody with the FITC-conjugated rabbit anti-mouse IgG and Texas-Red-tagged aptamers R1 (negative control), R3, R5 and R11 in RBSDV-free and RBSDV-infected SBPH midguts are presented in green and red signals, respectively. Bar = 100 μm.

To verify whether the selected aptamers could be used to detect virus in vivo, the Texas-Red-labeled putative aptamers R3, R5 and R11 were tested for detection of the RBSDV P10 protein in the midgut of the RBSDV-infected SBPH by immunofluorescence. The results confirmed the ability of selected aptamers as fluorescent probes in detecting the RBSDV P10 in SBPH midguts. To compare the virus localization detected by aptamers and the anti-RBSDV P10 mAB, the RBSDV localization was detected by Texas-Red fluorescent aptamers and RBSDV P10 mAB coupled with FITC-labeled rabbit anti-mouse IgG in RBSDV-infected SBPH midguts under the confocal microscope (Figure 8, each panel was cropped and grouped to allow more convenient comparison. For uncropped figures, please refer to Supplementary Figure S5). The merger of green and red fluorescence in the RBSDV-infected SBPH midgut confirmed the specificity and potential application of Texas-Red-tagged aptamers R3, R5 and R11 as fluorescent probes for detection of RBSDV P10 protein. The Texas-Red fluorescence-labelled aptamer R1 (negative control) did not bind to the RBSDV P10 in the SBPH midgut. These results indicate that the three novel DNA aptamers can detect virus in situ and can be used as alternative probes of the anti-RBSDV P10 mAB.

## 4. Discussion

Although aptamers have been proven to be applicable detection probes for a few animal viruses, the application of aptamer screening in virus detection was hindered by the complexity of the SELEX process and the unpredictable screening outcome. In order to establish a standardized SELEX process and screen out a specific aptamer for RBSDV P10 protein efficiently, the SELEX process was improved by optimizing the ePCR in SELEX and setting-up a close monitoring of the SELEX process, which would benefit similar selections of aptamers for plant virus protein detection in the future.

To conduct a successful SELEX, the high efficiency of ePCR is most crucial [26,42]. The equal amplification of each strand in the eluted aptamer library is the prerequisite for an efficient SELEX. The loss of diversity due to PCR bias would be greatly minimized and each putative aptamer in this library would be exploited equally in subsequent rounds of amplification. The process of emulsion creation, ePCR composition and DNA recovery method were firstly improved to standardize the emulsion PCR in SELEX in this study. There are several reported methods to create the emulsion by either vigorous mixing using a tissue lyser [22,43] or vortexing [23]. The uniformity of the emulsion created by a tissue lyser or vortexing was compared by observation using a light microscope. The vortexing method created a more uniform emulsion than vigorous mixing using a tissue lyser. Additionally, the organic phase was tested for thermal stability in emulsification [22,23]. As for optimization of the ePCR composition, the MgCl_2_ concentration was found to play a crucial role. For efficient amplification of ssDNA templates in the emulsion, the MgCl_2_ concentration between 3.5 and 4 mM was optimal for the ePCR when the aqueous phase was emulsified in the organic phase. This is contrary to the 1.5 mM MgCl_2_ that has been generally used in the conventional PCR. The importance of increasing the concentration of MgCl_2_ in ePCR has not been reported previously. It turned out to be the most important factor for the effective ePCR in the aptamer amplification. This might be due to the significantly higher (10 times) concentration of primers used in ePCR and the DNA primers competed for Mg^2+^ ions. Thus, primer DNA reduced free Mg^2+^ ions available for Taq polymerases, hindering the ePCR process. To further optimize ePCR, BSA was introduced to protect DNA polymerases from being trapped in the intermediate layers and boost PCR efficiency [23]. BSA was confirmed to be useful and a very small amount (0.01 mg/mL) was sufficient for efficient ePCR. The recovery of amplified dsDNA from the emulsion was also improved. Instead of using the diethyl ether or isobutanol for emulsion breaking [39], water-saturated isobutanol was used to protect the aqueous phase from the hygroscopicity of pure isobutanol during phase separation. With the use of the QIAquick PCR Purification Kit after emulsion breaking, the recovery of amplified DNA could be readily completed within 10 min.

A close monitoring of the output DNA yield and the output/input DNA ratio during SELEX provided valuable information on the enrichment of putative aptamers and standardized the whole screening process. In a typical SELEX process, putative aptamers are enriched when the output/input DNA ratio first declines and then starts to increase with an output DNA yield of more than 3 μg, despite continuously increased stringency parameters throughout SELEX rounds. In this study, these stringency parameters were adjusted based on the monitoring of the output/input DNA ratio to favor the enrichment of high affinity aptamers, while ensuring adequate output yield of DNA from ePCR in each round.

As for selected aptamer candidates for the RBSDV P10 protein detection, it is not possible to directly predict positive aptamers based on their sequence similarities by phylogenetic analysis. Thus, ELONA and aptamer-based dot-blot ELISA were conducted to verify the potential application of selected putative aptamers. The higher affinity of aptamers in ELONA did not correlate with high specificity in the aptamer-based dot-blot ELISA. This is probably due to the insufficient negative selection. In this study, the DNA library was negatively selected against freshly equilibrated Ni-NTA magnetic beads to reduce non-specific aptamers that interact with Ni^2+^ ions or the beads matrix. However, this process was unable to remove aptamers that potentially interacted with other SBPH proteins. An alternative could be using RBSDV-free SBPH proteins immobilized on beads as bait to remove non-specific aptamers. Additionally, since both the forward and reverse strands were pooled in the SELEX process, both strands from the T-A cloning constructs should be verified. The predicted folding of an F-aptamer and its antisense R-aptamer was similar in our preliminary analysis. However, the specificity of the binding to the target turned out to be very different, as shown in the F3/R3, F5/R5 and F11/R11 pairs. Aptamers R3, R5 and R11 are not the strongest binders to the RBSDV P10 protein but they shared good specificity towards the RBSDV P10 protein, thus making them the preferred aptamers. The finding of higher specificity in antisense R-aptamers and the irrelevance between the affinity and the specificity in aptamer enrichment was consistent with a previous report [38].

This is the first time that aptamers were applied in immunofluorescent labeling for a plant virus insect vector. The detection of the RBSDV P10 protein by Texas-Red-tagged aptamers R3, R5 and R11 was proven to be applicable in the immunofluorescent labeling. Moreover, the fluorescent signals from aptamers (1 μg/mL) R5 and R11 surpassed that of the mAB (2 μg/mL), as shown by stronger signals and lower background fluorescence. This may be due to the relatively smaller molecular weight of aptamers (~30 kDa), as compared to that of mAB IgG (~150 kDa) or smaller size of aptamers (3 nm) compared to that of mAB (10–15 nm) as reported previously [19]. Thus, the steric hindrance of aptamers was lower, making target molecules more accessible to aptamers than to antibodies. The FITC signal from mAB and Texas Red signal from aptamers did not always match perfectly in every pixel, especially in Figure 8 R11-merged. This might be due to the difference in the interaction force of mAB and aptamers to the target, as mAB binds to the target mainly through van der Waals forces, hydrogen bonding and hydrophobic interaction, while aptamers bind to target mainly through electrostatic interactions. The difference in the form of interaction could produce a slight difference in the immunofluorescent labeling. Another explanation may be that the epitopes of the mAB and aptamers recognition sites are not identical.

In this study, three ssDNA aptamers were selected from the optimized emulsion PCR-based SELEX as specific and sensitive probes for the detection of the RBSDV P10 protein both in extracted insect proteins in vitro and in the midgut of the RBSDV-infected SBPH in situ. The sensitivity and specificity of these three aptamers were comparable to the anti-RBSDV P10 protein mAB based on their performance in the dot-blot ELISA and the immunofluorescent localization. These ssDNA aptamers can be modified and synthesized chemically and further developed into potential probes for detection of RBSDV. Based on the successful screening of aptamers against the RBSDV P10 protein, the emulsion PCR-based SELEX could be applied to the selection of aptamers for other plant virus proteins.

Aptamers have been proven to be more sensitive and produced less nonspecific background in immunohistochemistry for clinical diagnosis [44]. Although aptamers are yet to be used widely, the advantages of aptamers over antibodies such as reduced batch-to-batch variations, commercial availability and excellent extensibility will help aptamers to gain more acceptance in virus–vector interaction studies in the future. Although SELEX requires advanced skills and close manipulation of the parameters throughout the process, the selection of aptamers for detection of viral protein is more convenient and economical, as compared to the mAB production, with a lesser requirement of equipment and could be completed within one week. The successful selection of aptamers R3, R5 and R11 against the RBSDV P10 protein has proven that ssDNA aptamers are viable alternatives to antibodies. The advantages of aptamers, such as easy synthesis, easy modification and low requirements for storage, could pave the way for the commercial use of aptamers as rapid detection and quantification tools in studies of plant virus–vector interactions.

## 5. Conclusions

In conclusion, three ssDNA aptamers R3, R5 and R11 were selected by the emulsion PCR-based SELEX against the purified RBSDV His-P10 protein and verified by ELONA. The specificity in detection of the RBSDV P10 protein in extracted proteins and in the infected midgut tissues of SBPH was verified in dot-blot ELISA and the immunofluorescent localization was confirmed by the anti-RBSDV P10 protein mAB. The aptamer selection method by emulsion PCR-based SELEX was improved and standardized in this study to assist researchers in future selection of aptamers to localize plant virus proteins in transmission vectors.

## Figures and Tables

**Table 1:** Changes of parameters in screening aptamers against RBSDV His-P10 protein by SELEX from rounds 1 to 10.

Round	Input DNA (Folded Aptamers)		① P10-Beads Complex		② Binding Time	③ Wash Time	③ Wash Volume	④⑤ ePCR Cycles	⑥ Monitor Index		
	μg	pmol	Beads (μL)	P10 (μg)	(min)		(μL)		Output DNA (μg)	pmol	Output/Input Ratio
**1**	35.7	1000	30	30	90	1	500	20	12.800	179.2	0.18
**2**	3	42	15	15	60	1	500	20	10.070	141.0	3.36
**3**	2	28	15	15	45	2	500	20	3.010	42.1	1.51
**4**	2	28	15	15	45	2	500	20	4.385	61.4	2.19
**5**	2	28	15	15	45	2	500	20	4.175	58.5	2.09
**6**	2	28	15	15	45	2	1000	20	7.665	107.3	3.83
**⑦ Negative Selection I**	2	28	20	0	60						
**7**	2	28	15	15	45	2	1000	20	12.515	175.2	6.26
**8**	2	28	15	15	30	3	1000	20	14.105	197.5	7.05
**⑦ Negative Selection II**	1	14	20	0	60						
**9**	1	14	15	15	20	3	1000	15	12.080	169.1	12.08
**10**	0.5	7	5	5	15	3	1000	10	4.105	57.5	8.21

For consistency of description of selection procedures in Figure 1 and Table 1, the serial numbers and footnotes were added for better elaboration of the selection process. ① Target protein (RBSDV His-P10) immobilized to Ni^2+^ Mag-beads; ② incubation/binding of aptamers to P10-Ni^2+^ Mag-beads; ③ weakly bound aptamers removed; ④ bound aptamers eluted as PCR templates; ⑤ eluted aptamers amplified by water-in-oil emulsion PCR (ePCR); ⑥ amplified aptamers recovered; ⑦ nonspecific aptamers removed by empty beads (negative selection).

**Table 2:** The selection of 23 non-redundant dsDNA sequences (as templates for putative aptamers against the RBSDV His-P10 protein) from 50 T-A cloning constructs at SELEX round 10.

Serial No.	T-A Clone No.	Sequence (5′-3′)	nt
1	1	TGACACCGTACCTGCTCTAGATGAAGACTGATGATGCCTGTCAACGCGCGGAACCACTGGATCAAGACGGACCGGAACCGTGTTCACTGTCGACAAGCACGCCAGGGACTAT	118
2	3	TGACACCGTACCTGCTCTAGCGGTCTCGTGAATCTGCAGACGAATTTGTGTAATATGAGCGCATATGTATAACATTGCAGACAAGGTCGAGAGCGCGTAAGCACGCCAGGGACTAT	122
3	4	TGACACCGTACCTGCTCTGGGTGTGGAGATTGTGAGGGGAGGTGCTAGCATGGATAGAAAGTAAAGAGGAGGAAAGTGCGGAGTAAGGAGGCGGGGAAGCACGCCAGGGACTAT	120
4	5	TGACACCGTACCTGCTCTGACAGTCCTCGTACAAAGGTCGAGTCATCATACCCGCCGTAACCCCTTACGGGTCGAGCCAAGCACGCCAGGGACTAT	102
5	6	TGACACCGTACCTGCTCTCAGGGCAACACTATGACATAGGGAACTCTCGGAACACAGGGAGGCCTCCAGTACAATTCGGATGTGAACTGCAAGCACGCCAGGGACTAT	114
6	11	TGACACCGTACCTGCTCTAGCACGGCACCCATGGGCAGTATACCGTCCCCCGCGAAAGACGGGCCGCTGCGGTAAGCACGCCAGGGACTAT	97
7	13	TGACACCGTACCTGCTCTACCCCGCATTATCCTTATAACCCGATAGAATAAACGACGAGGTCGGCGAAAGACCGAAGGCGGTGCAGGGCCAAGCATGCAAGCACGCCAGGGACTAT	122
8	15	TGACACCGTACCTGCTCTAGCCACCTTCGTAGACACCATATCAGAAAGAGATACCCAGGAGGCGCCCCCGTGTGCGAACGAAGGGCGATTGTAACGAAGCACGCCAGGGACTAT	120
9	16	TGACACCGTACCTGCTCTGGGCCGTTGATTCCAACCCTTTATGGCGGCGTGCGAAAGACACGATCAACGCGCAGCCCCAAGCACGCCAGGGACTAT	102
10	17	TGACACCGTACCTGCTCTGCCGTAGTGATCCTCCTTAACAGTGACCTATAGATCGTTCGTCGTATTTGCTTTGACAATCCTTCTCAGATACTCTCCCGAAGCACGCCAGGGACTAT	122
11	20	TGACACCGTACCTGCTCTAGTCGTGGTCATGCAGCACGGAAGCGTAAGACTTCCCGCTGGACGGTCGGTAAGACGGTAAATGACTCCGCAAGCACGCCAGGGACTAT	113
12	21	TGACACCGTACCTGCTCTCTACCAGCTCCGACGATCCAGTATTGTGAACCCCGCGCGGTAGCGTTCTCGTCTTCGTGGAGTGGAACGCAAGCACGCCAGGGACTAT	112
13	22	TGACACCGTACCTGCTCTGCCCGTAGACAAAGTCCGCCCCGAAATCGCAAGACTGCAAGCGAAAGACTAAAGCGATCGAATAAGATCCGATTCAGGGGAAAGCACGCCAGGGACTAT	123
14	24	TGACACCGTACCTGCTCTGCCGTAGTGATCCTCCTTAACAGTGACCTATAGATCGTTCGCCGTATTTGCTTTGACAATCCTTCTCAGATACTCTCCCGAAGCACGCCAGGGACTAT	122
15	25	TGACACCGTACCTGCTCTGGGCCGTTGATTCCAACCCATTATCGCGGCGTGCGAAAGACACGATCAACGCGCAGCCCCAAGCACGCCAGGGACTAT	102
16	26	TGACACCGTACCTGCTCTGGGTCAGCGAAAGACTACCCCCGTGGCGCGCGGAAGACGCGCCCAGCAGGACCAGCCGAACACCCCAAGCACGCCAGGGACTAT	108
17	27	TGACACCGTACCTGCTCTAGGGAGTCGATGTTCCCTGCTCACGACATGGTCCGCGGAAGACGGACAGGACATCCCGAGAAGCGAGCGAGCATCTCGCTAAGCACGCCAGGGACTAT	122
18	29	TGACACCGTACCTGCTCTACCTGGATGCGCGTTAGACGCTGTTGAATAATCATCGCAAAGAGTCCAGGTACGCAAGGTTATGGACAGTGTGCCAAGCACGCCAGGGACTAT	117
19	32	TGACACCGTACCTGCTCTAGAACGCAGCTCAAAAGCTCGCCCAGCACCCGAAAAGAGGTGCGGCCGTCTGAGGGAAGCAGCGCAGAACCCCGAAGCACGCCAGGGACTAT	116
20	34	TGACACCGTACCTGCTCTGGAGACGAAGAGATAAGGCAGAAGTTGAGAAGATGGGATGAGGGCCGGGAAGCACGCCAGGGACTAT	91
21	36	TGACACCGTACCTGCTCTACCCCGCATTATCCTTATAACCCGATAGAATAAATGACGAGGTCGGCGAAAGACCGAAGGCGGTGCAGGGCCAAGCATGCAAGCACGCCAGGGACTAT	122
22	38	TGACACCGTACCTGCTCTGGGCCGTTGATTCCAACCCTTTATCGCGGCGTGCGAAAGACACGATCAACGCGCAGCCCCAAGCACGCCAGGGACTAT	102
23	45	TGACACCGTACCTGCTCTGGGCGGTGCGTCGCTTTGCTGAAGCCAGATTGGTCTTTGACAGAGATCAGACAGAACTGTTCCAGAAAGCGAGGGAAGCACGCCAGGGACTAT	117

The primer set flanking the N80 ssDNA library was adopted from Schütze’s previous paper [38] to be devoid of hairpin, dimer, and heterodimer-forming sequences. The forward primer region (blue) and reverse primer region (red) of the screened-out aptamers are highlighted in Table 2, respectively.

## Supplementary Materials

The following are available online at https://www.mdpi.com/1999-4915/12/11/1239/s1, Figure S1: Induction of the RBSDV P10 protein by different protein expression constructs. Figure S2: Expression and purification of the RBSDV His-P10 protein. Figure S3: Optimization of emulsion creation process and BSA concentration for ePCR. Figure S4: Uncropped figures (all 46 panels) of Figure 7. Figure S5: Uncropped figures (all 32 panels) of Figure 8.
