# Supplementary material for: Selection of DNA Aptamers for Subcellular Localization of RBSDV P10 Protein in the Midgut of Small Brown Planthoppers by Emulsion PCR-Based SELEX
Source: Viruses. 2020 Oct 30;12(11):1239. doi: 10.3390/v12111239 (PMC7692627; doi:10.3390/v12111239)
Supplement: Supplementary file 1 [file viruses-12-01239-s001.pdf]

Supplementary Figures

Selection of DNA aptamers for subcellular localization of RBSDV P10 protein in the midgut of small brown planthoppers by emulsion-PCR-based SELEX

Haoqiu Liu[1,2], Yijun Zhou[3], Qiufang Xu[3*] and Sek-Man Wong[1,2,4*]

1 Department of Biological Sciences, National University of Singapore, Singapore 117543, Singapore;
liuhaoqiu@u.nus.edu

2 National University of Singapore (Suzhou) Research Institute, Suzhou 215123, China

3 Institute of Plant Protection, Jiangsu Academy of Agricultural Sciences, Key Laboratory of Food Quality and Safety of Jiangsu Province, State Key Laboratory Breeding Base, Nanjing 210014, China

4 Temasek Life Sciences Laboratory, 1 Research Link, Singapore 117604, Singapore

* Correspondence: xuqiufang@jaas.ac.cn; Tel.: +86-25-84390394; Fax: +86-25-84390391

* Correspondence: dbswsm@nus.edu.sg; Tel.: +65-65162976; Fax: +65-67792486

Figure S1A.

Induction of RBSDV P10 protein by different protein expression constructs

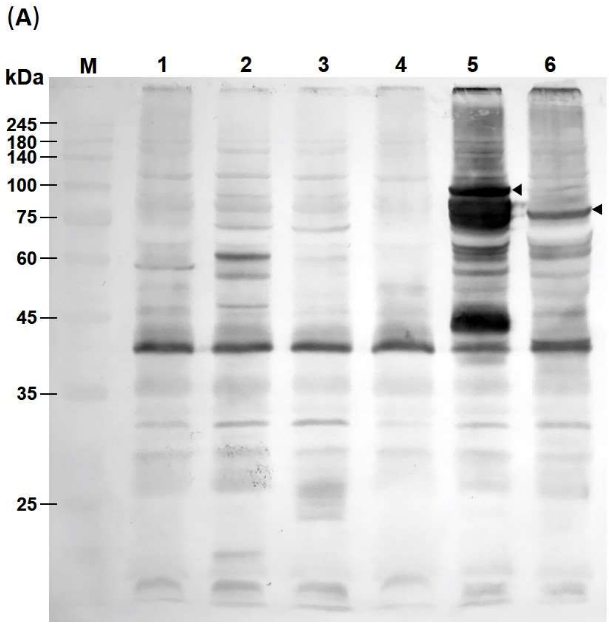

Figure S1A. Induction of RBSDV P10 protein by different protein expression constructs (Lane 1: pET-15b-His-P10; Lane 2: pET-28m-His-SUMO-P10-His; Lane 3: pET-32a-TrxA-His-Stag-P10; Lane 4: pET-42b-GST-His-Stag-P10-His; Lane 5: pMAL-c2X-MBP-P10; Lane 6: pGEX-6p-1-GST-P10, respectively) in *E. coli* Rosetta (DE3) cells with 0.1 mM IPTG at 30 oC for 6 h. Total cell protein after induction were separated by 12.5 % SDS-PAGE and proteins were transferred to nitrocellulose membrane (0.45 µm) and detected by anti-RBSDV P10 monoclonal antibody. Arrowheads represent MBP- or GST-tagged P10 protein.

Figure S1B.

Induction of RBSDV P10 protein by different protein expression constructs

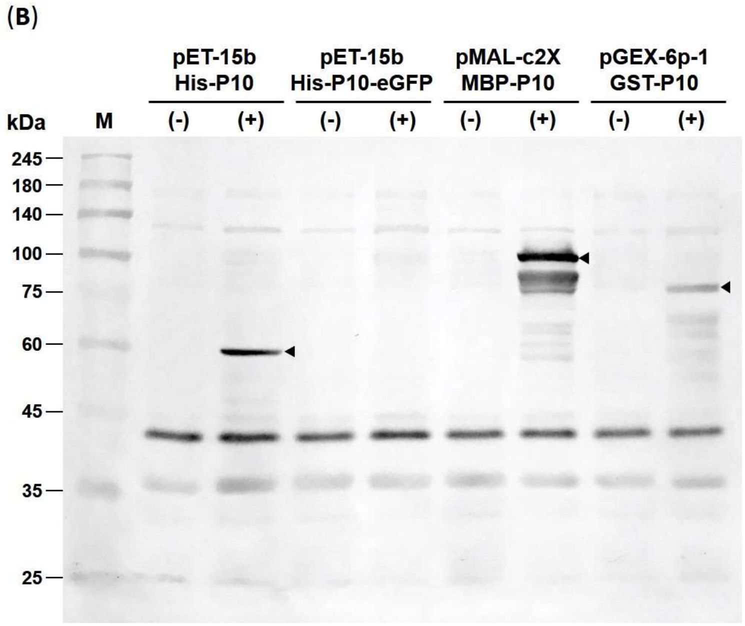

Figure S1B. Induction of RBSDV P10 protein by different protein expression constructs (pET-15b-His-P10, pET-15b-His-P10-eGFP, pMAL-c2X-MBP-P10 and pGEX-6p-1-GST-P10, respectively) in *E. coli* Rosetta (DE3) cells with 0.1 mM IPTG at 16 ^{o}C for 18 h. Samples before (-) and after (+) induction were separated by 12.5 % SDS-PAGE and proteins were transferred to nitrocellulose membrane (0.45 μm) and detected by anti-RBSDV P10 monoclonal antibody. Arrowheads represent His-, MBP- or GST-tagged P10 protein.

Figure S2.
Expression and purification of RBSDV His-P10 protein.

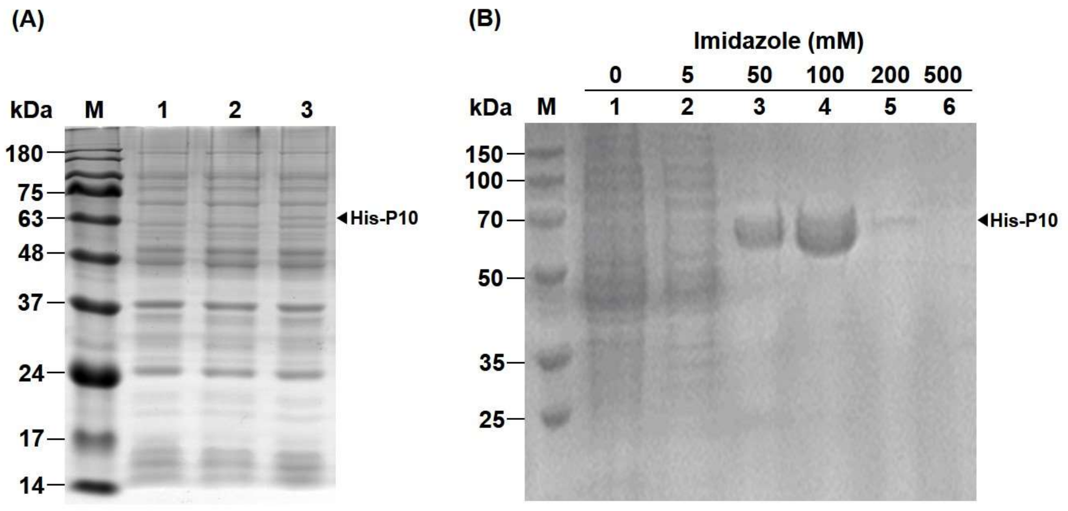

Figure S2. Expression and purification of RBSDV His-P10.
(**A**) IPTG induction of RBSDV His-P10 protein in BL21-RIPL(DE3) CodonPlus *E. coli* cells. BL21-RIPL(DE3) CodonPlus *E. coli* cells transformed with pET-15b empty vector or pET-15b-P10 construct after induction with 0.6 mM IPTG at 16 ^{o}C for 16 h were lysed and separated by 12.5 % SDS-PAGE, stained by Coomassie brilliant blue R250. Lysate of *E. coli* cells transformed with pET-15b empty vector after 0.6 mM IPTG induction (as negative control; Lane 1); Lysate of *E. coli* cells transformed with pET-15b-P10 before (Lane 2) and after (Lane 3) IPTG induction.
(**B**) Purification of RBSDV His-P10 by IMAC with Ni-NTA resins. Fractions collected during IMAC were separated by 12.5 % SDS-PAGE. Flow-through unbound proteins (Lane 1); Imidazole eluents, 5 mM (Lane 2); 50 mM (Lane 3); 100 mM (Lane 4); 200 mM (Lane 5) and 500 mM (Lane 6).

Figure S3.

Optimization of emulsion creation process and BSA concentration for ePCR.

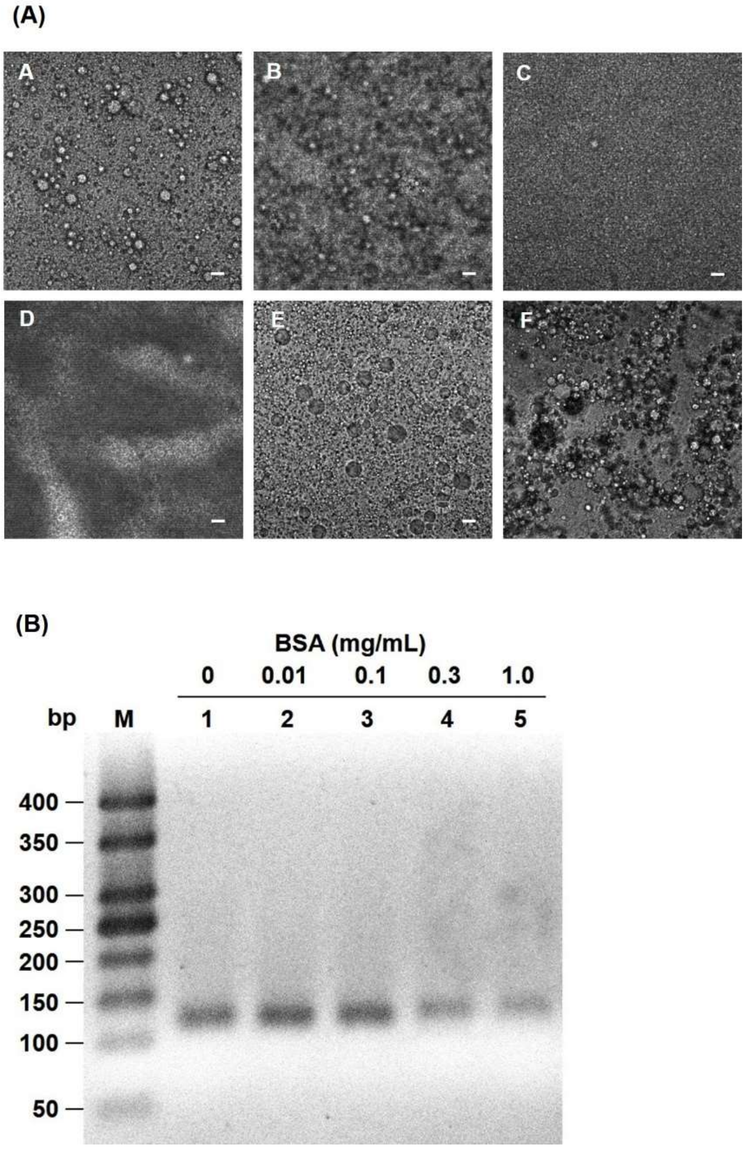

Figure S3. Optimization of emulsion creation process and BSA concentration for ePCR.

(**A**) Comparison of emulsification methods using tissue lyser versus vortexing at 4 °C. Tissue lyser at 30 Hz for 20 sec (A) and 40 sec (B), respectively. Tissue lyser at 60 Hz for 20 sec (C) and 40 sec (D), respectively. Vortexing at 3000 rpm for 5 min at 4 °C (E) and 25 °C (F), respectively. Please note the uniform emulsified droplets in panel E. Bar = 10 µm. Each panel was cropped and grouped to allow more convenient comparison. For uncropped figures, please refer to the supplementary Figure S1.

(**B**) Optimization of BSA concentrations of 0, 0.01, 0.1, 0.3 and 1.0 mg/mL, respectively. Purified DNA from 20 cycles of ePCR with gradient BSA concentrations and 4 mM $MgCl_2$ separated by agarose gel electrophoresis, stained by ethidium bromide (0.5 µg/mL).

Figure S4.

Uncropped figures (all 46 panels) in Figure 7. Detection of RBSDV P10 protein with 46 biotinylated putative aptamers by Dot-ELISA.

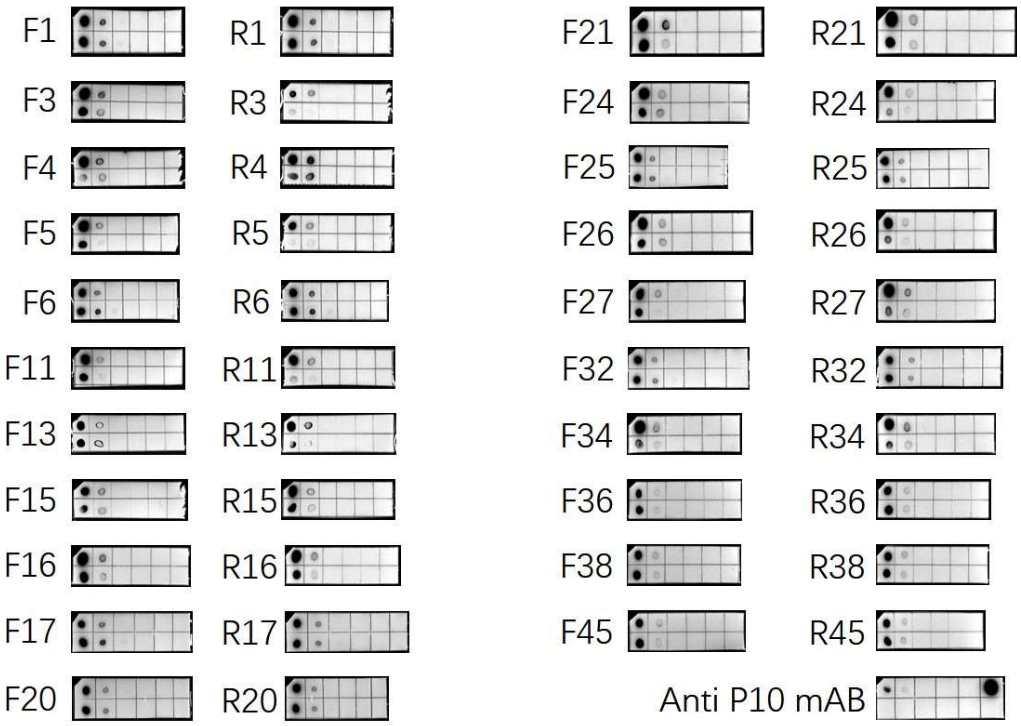

Figure S4. The cytosolic protein extracted from RBSDV-infected SBPH (upper row) and RBSDV-free SBPH (lower row) were diluted 10^2x, 10^3x, 10^4x, 10^5x and 10^6x, and 1 μL of diluted protein solution was blotted onto the NC membrane (from left to right). The rightmost column from "F1"/"R1" to "F45"/"R45" was blank control. The upper right block of "Anti P10 mAB" was blotted with 1 μL undiluted cytosolic protein fraction extracted from RBSDV(+) SBPH as a positive control to the serial dilutions.

Figure S5.

Uncropped figures (all 32 panels) for Figure 8. Subcellular localization of Texas-Red-tagged aptamers R1 (negative control), R3, R5 and R11 with anti-RBSDV P10 protein monoclonal antibody (mAB) in the midgut of RBSDV-infected small brown planthoppers (SBPH).

Panels are arranged as mAB (green), phase contrast (white), aptamer (red) and merged.

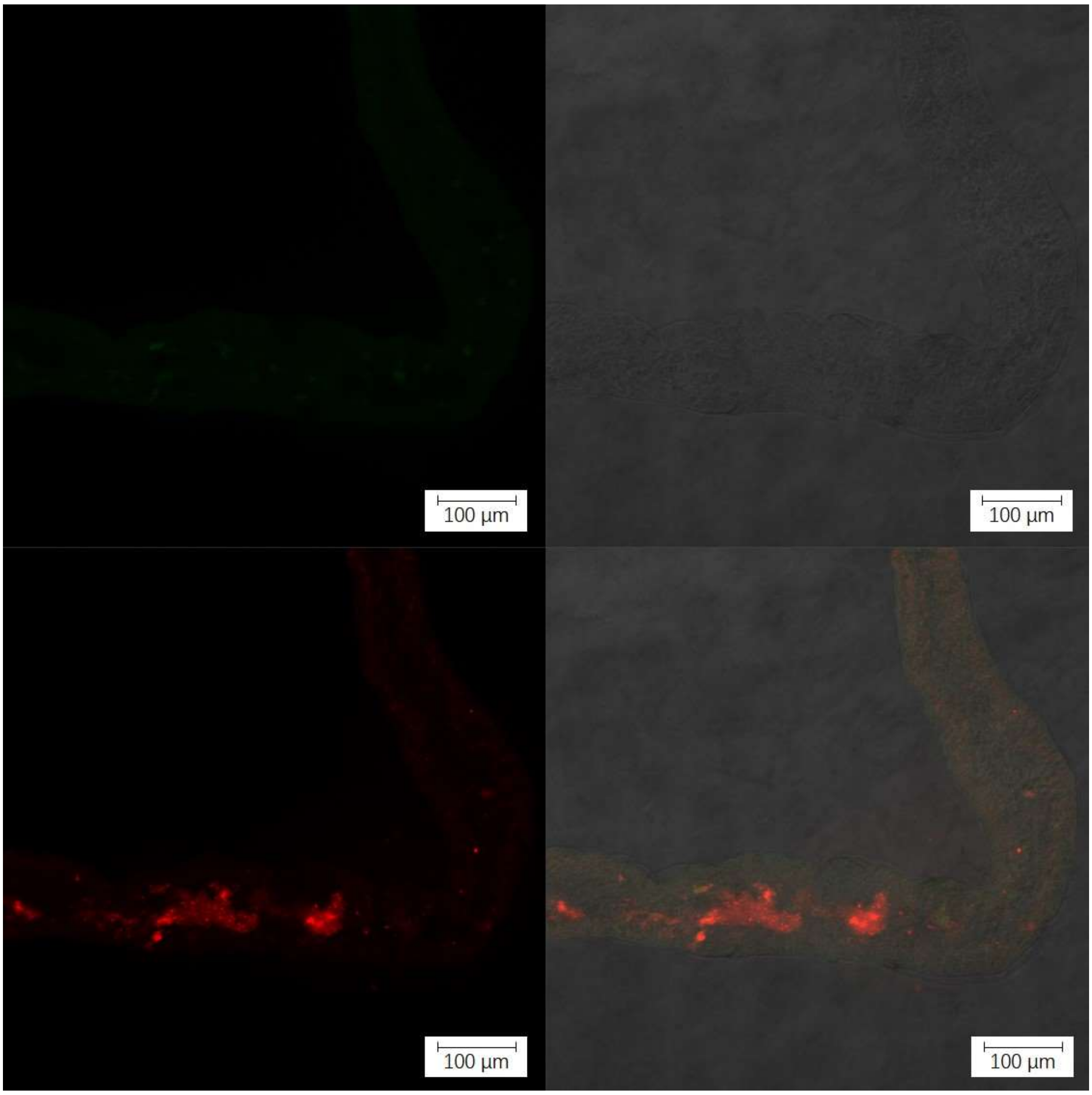

Figure S5A. P10 mAB (green) and Texas-Red-tagged R1 (red) aptamer in RBSDV-free SBPH midgut.

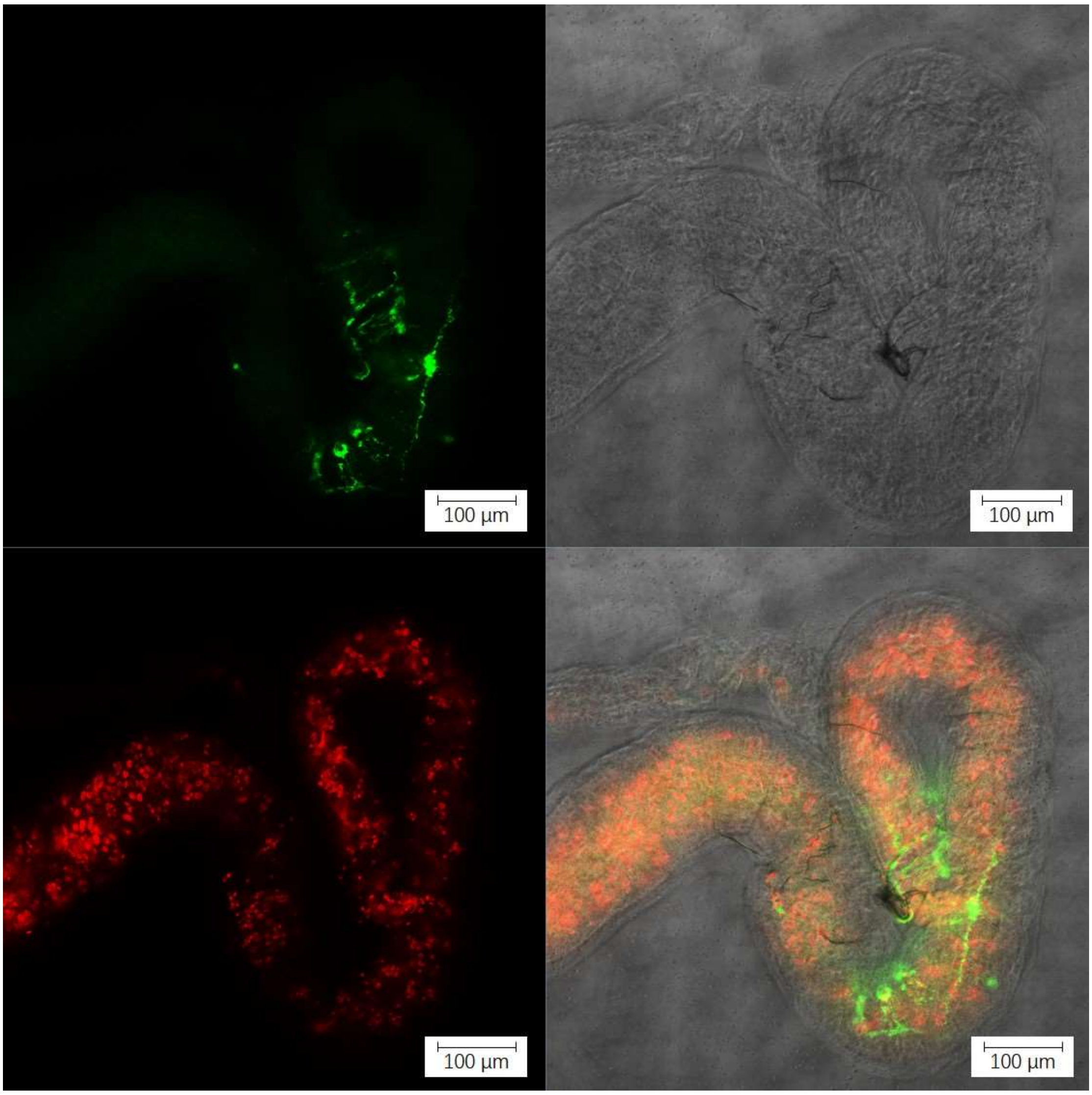

Figure S5B. P10 mAB (green) and Texas-Red-tagged R1 (red) aptamer in RBSDV-infected SBPH midgut.

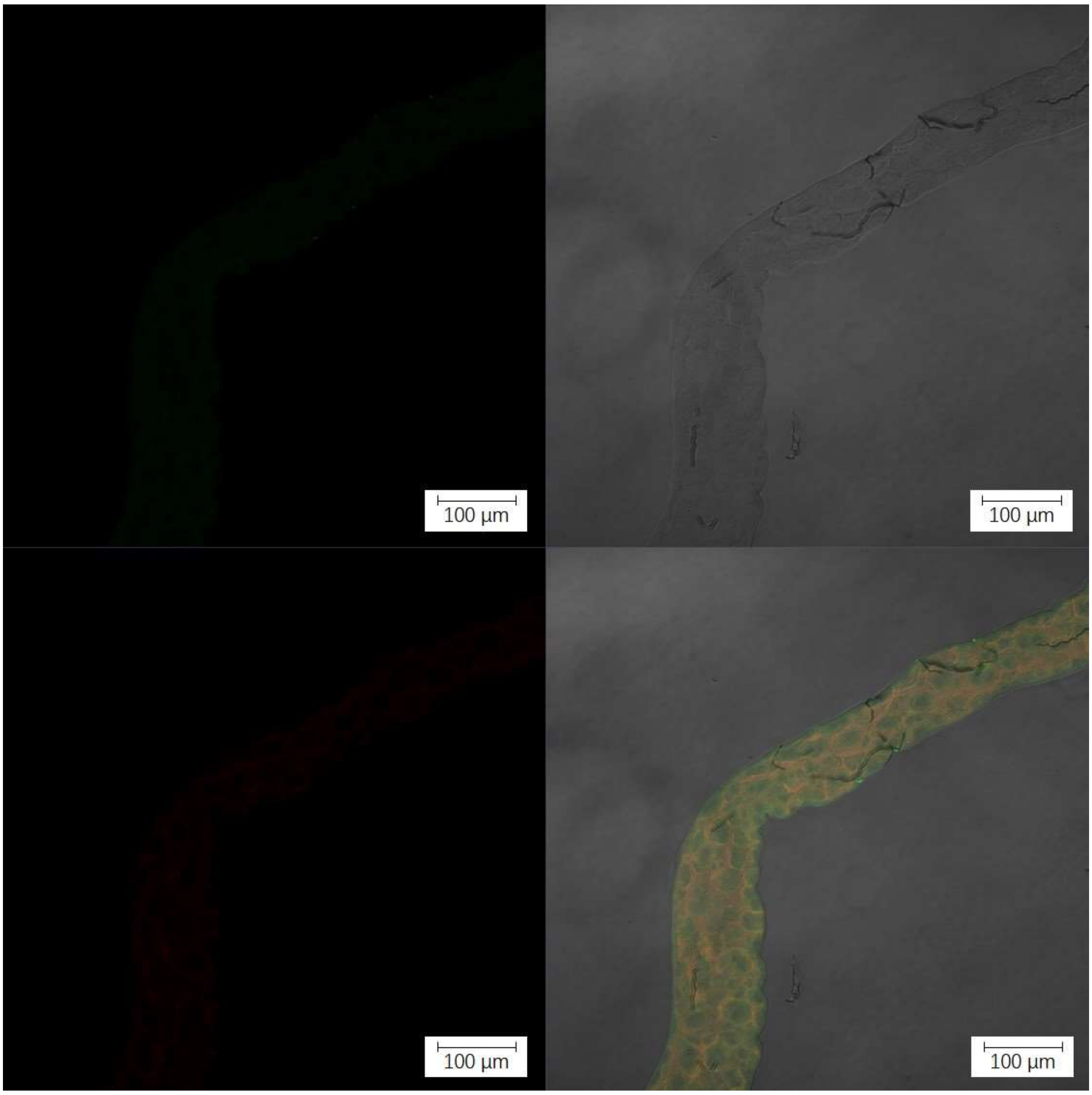

Figure S5C. P10 mAB (green) and Texas-Red-tagged R3 (red) aptamer in RBSDV-free SBPH midgut.

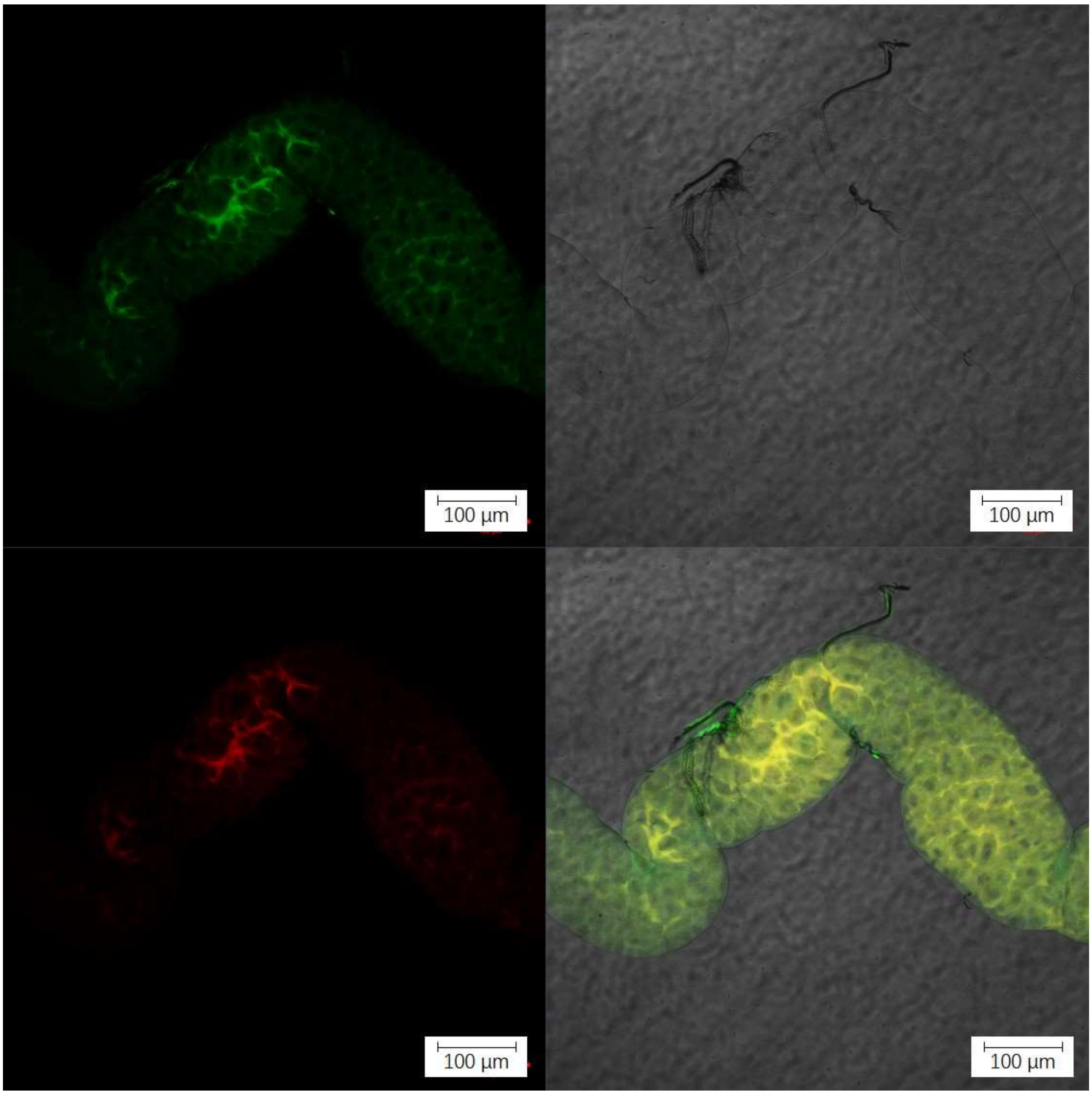

Figure S5D. P10 mAB (green) and Texas-Red-tagged R3 (red) aptamer in RBSDV-infected SBPH midgut.

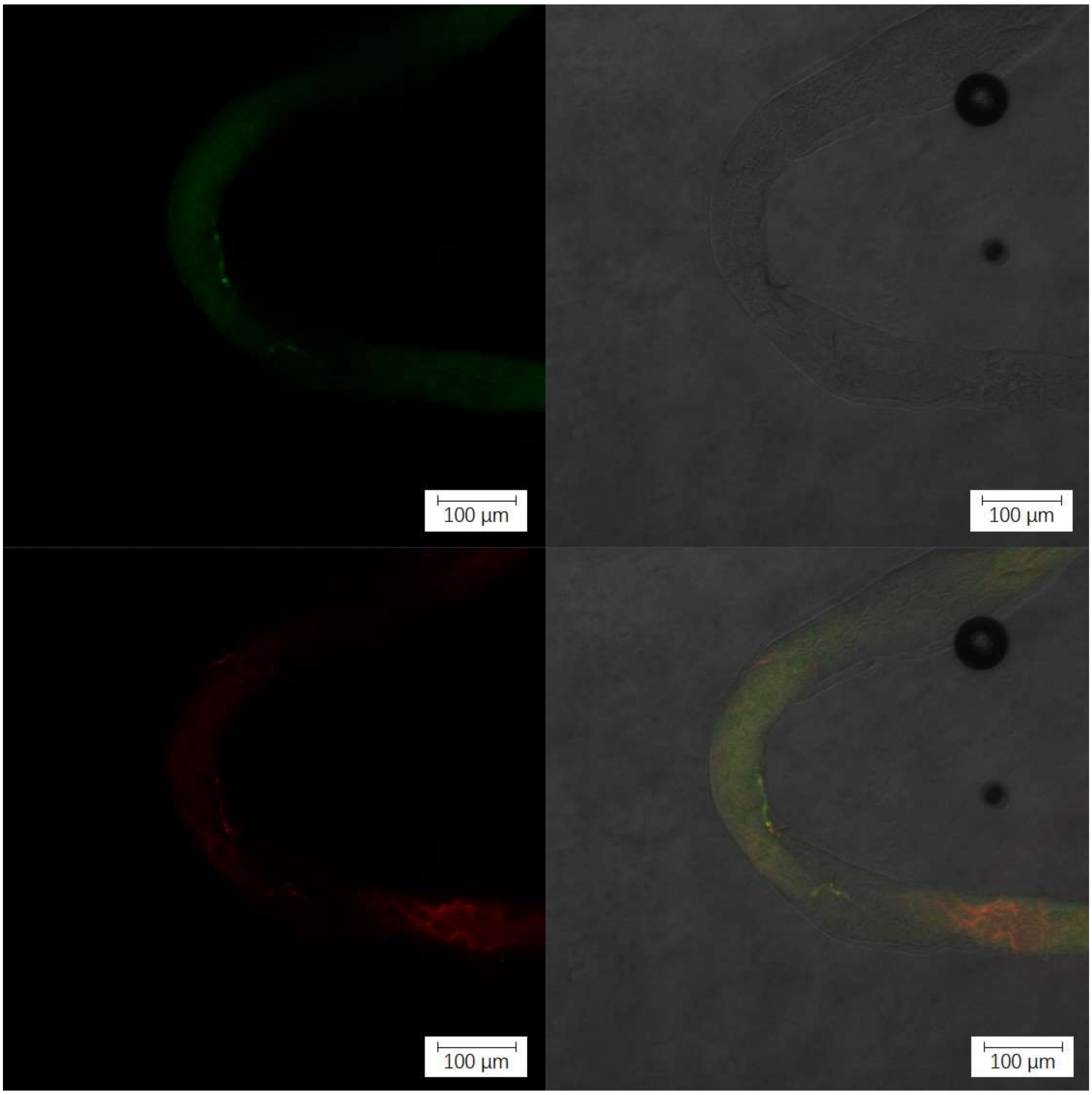

Figure S5E. P10 mAB (green) and Texas-Red-tagged R5 (red) aptamer in RBSDV-free SBPH midgut.

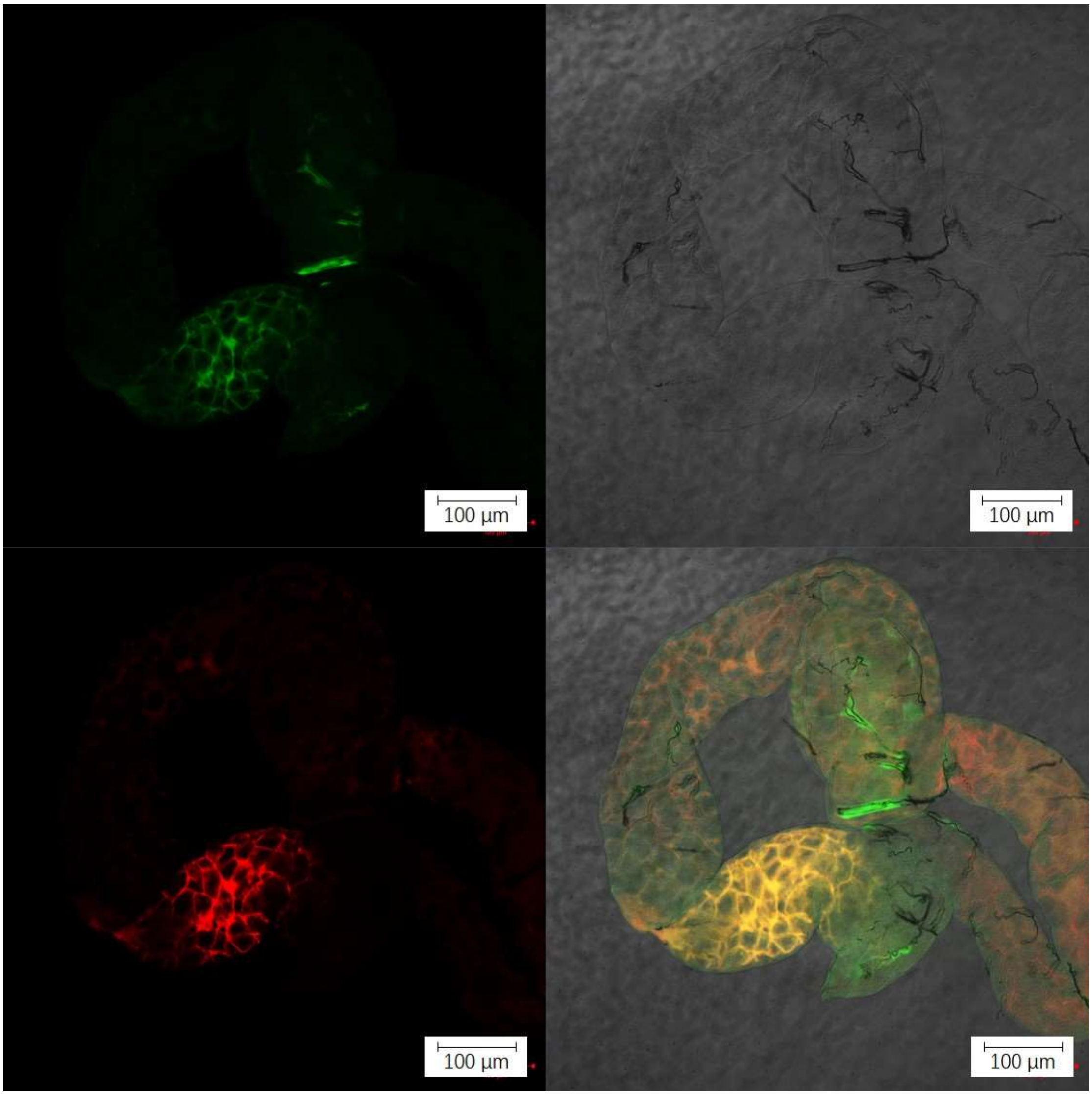

Figure S5F. P10 mAB (green) and Texas-Red-tagged R5 (red) aptamer in RBSDV-infected SBPH midgut.

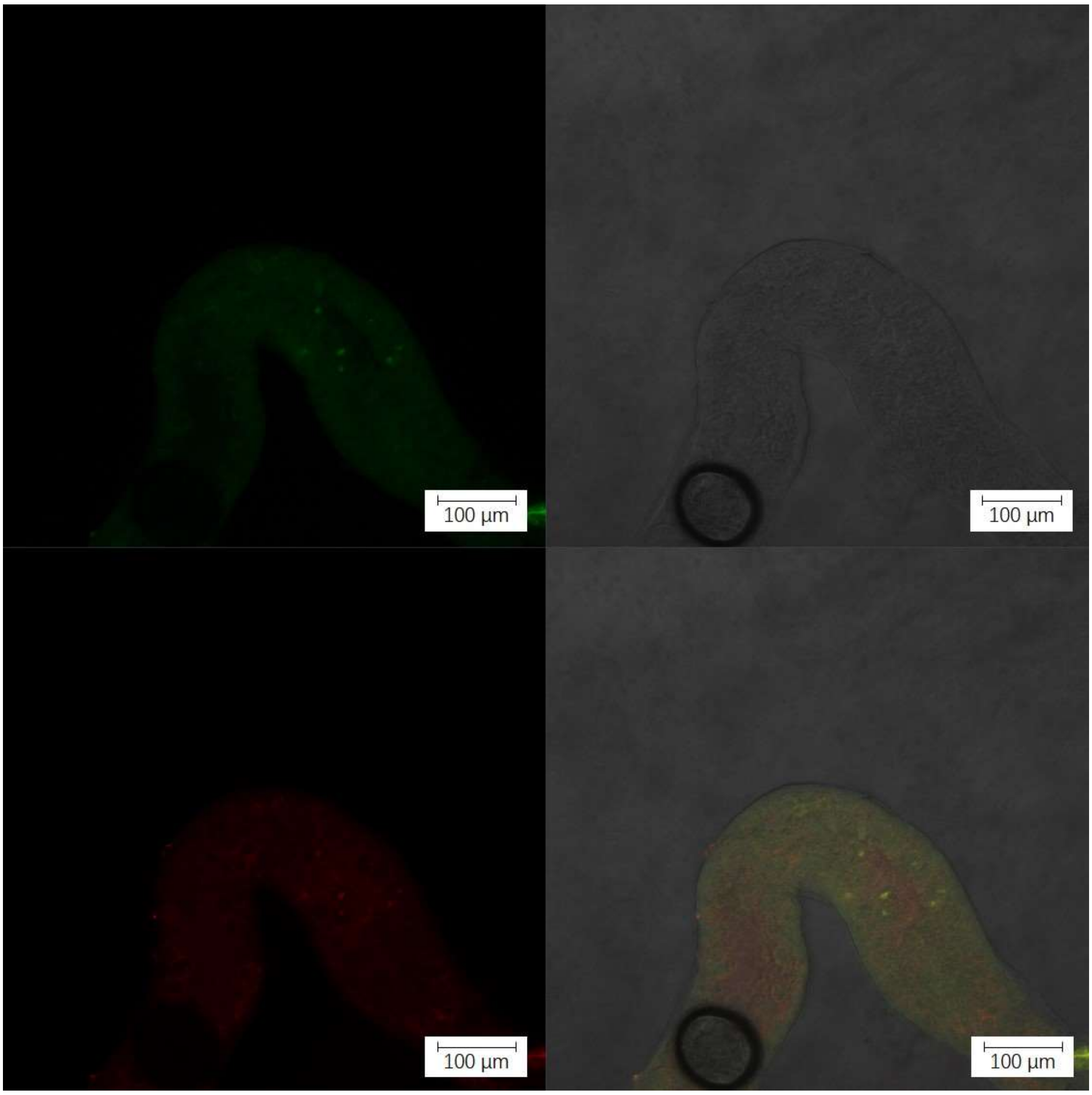

Figure S5G. P10 mAB (green) and Texas-Red-tagged R11 (red) aptamer in RBSDV-free SBPH midgut.

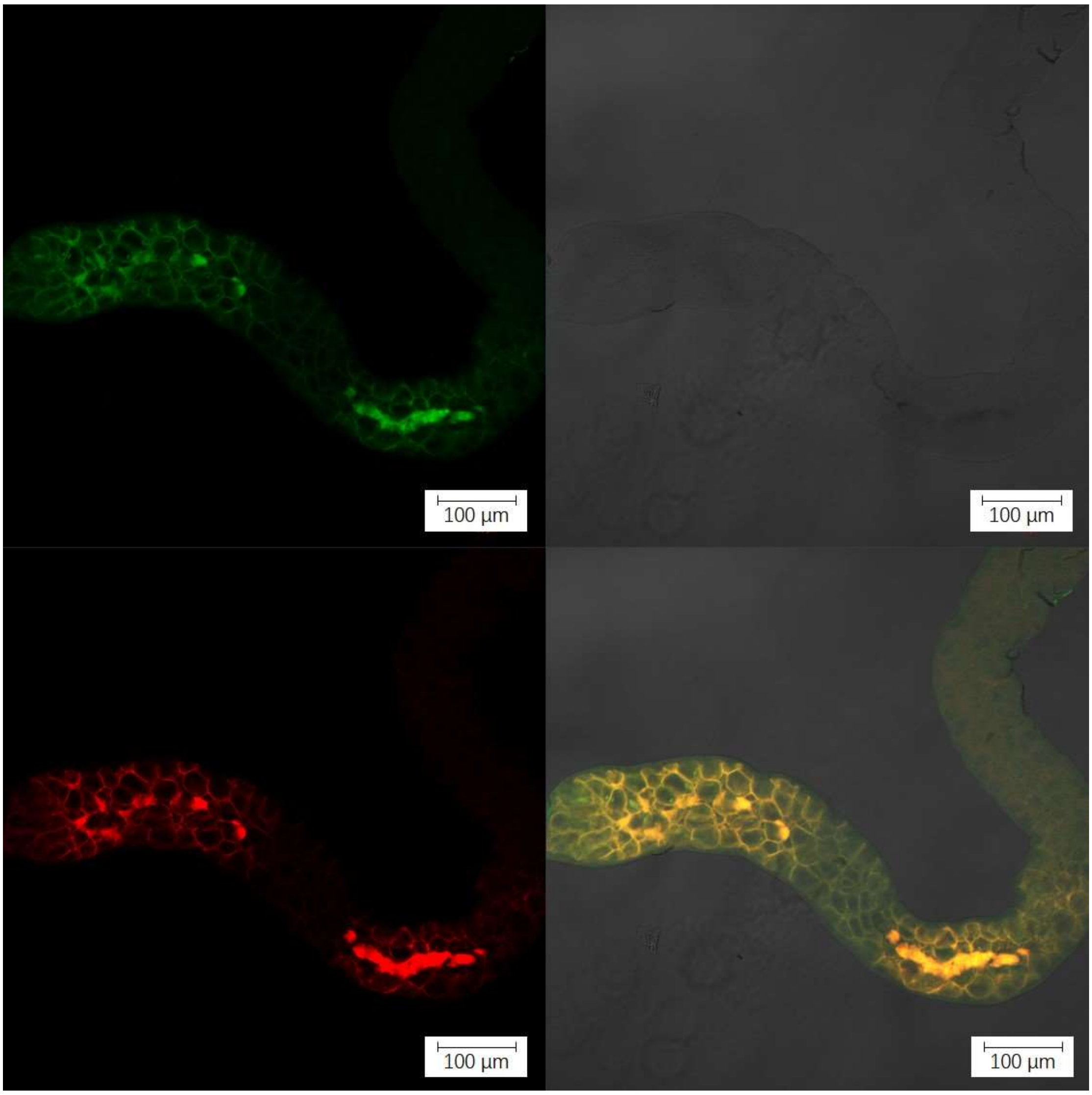

Figure S5H. P10 mAB (green) and Texas-Red-tagged R11 (red) aptamer in RBSDV-infected SBPH midgut.
